# Harnessing joint distraction for the treatment of osteoarthritis: a bibliometric and visualized analysis

**DOI:** 10.3389/fbioe.2023.1309688

**Published:** 2023-11-09

**Authors:** Liqing Peng, Runmeng Li, Shengxi Xu, Keyuan Ding, Yan Wu, Hao Li, Yong Wang

**Affiliations:** ^1^ Department of Orthopedics, First People’s Hospital of Shuangliu District, Chengdu, China; ^2^ School of Medicine, Nankai University, Tianjin, China

**Keywords:** bibliometrics, joint distraction, osteoarthritis, cartilage, visualization research

## Abstract

Osteoarthritis (OA) stands as a prevalent degenerative joint ailment, demanding immediate attention towards the development of efficacious therapeutic interventions. Presently, a definitive cure for OA remains elusive, and when conservative treatment modalities prove ineffective, resorting to a joint prosthesis becomes imperative. Temporary distraction emerges as a pivotal joint-preserving intervention in human OA patients, conferring both clinical amelioration and structural enhancements. Although extant clinical investigations exist, they are characterized by relatively modest sample sizes. Nonetheless, these studies furnish compelling evidence affirming that joint distraction engenders sustained clinical amelioration and structural refinement. Despite substantial strides in the last decade, a bibliometric analysis of joint distraction within the realm of osteoarthritis treatment research has been conspicuously absent. In this context, we have undertaken a comparative investigation utilizing bibliometric methodologies to scrutinize the landscape of joint distraction within osteoarthritis treatment. Our comprehensive analysis encompassed 469 scholarly articles. Our findings evince a consistent escalation in global research interest and publication output pertaining to this subject. The United States emerged as the frontrunner in international collaboration, publication count, and citation frequency, underscoring its preeminence in this domain. The journal “Osteoarthritis and Cartilage” emerged as the principal platform for disseminating research output on this subject. Notably, Mastbergen SC emerged as the most prolific contributor in terms of authorship. The identified keywords predominantly revolved around non-surgical interventions and joint arthroscopy procedures. This bibliometric analysis, augmented by visual representations, furnishes invaluable insights into the evolutionary trajectory of joint distraction as an osteoarthritis treatment modality spanning from 2003 to 2023. These insights will serve as a compass for the scientific community, facilitating further exploration in this promising domain.

## 1 Introduction

With an increasingly aging population and a rising prevalence of obesity, there is a burgeoning demand for interventions that preserve joints afflicted by osteoarthritis (OA) (Bierma-Zeinstra, 2019). As the predominant form of arthritic affliction, OA is principally distinguished by the gradual erosion of cartilaginous matrix and pathological alterations in other integral joint constituents, encompassing the emergence of osteophytes, hyperplasia of the synovial membrane, and the onset of oxidative stress and chondrocytes apoptosis (Chandran et al., 2019; Chang et al., 2019; Choi et al., 2019; Hunter et al., 2020; Zhu et al., 2022). The etiology of OA is predominantly linked to the processes of aging and the deleterious effects of aberrant mechanical stress on the chondrocytes microenvironment (Prieto-Alhambra et al., 2014). However, the articular cartilage, being a highly specialized tissue, lacks vascularization for nutritional sustenance, thereby conferring upon it a limited innate capacity for self-repair. The preservation of resilient chondrocytes within the cartilaginous milieu assumes paramount importance in the maintenance of joint vitality. In clinical practice, the management of early-stage OA predominantly hinges on non-pharmacological and pharmacological modalities. Non-pharmacological strategies chiefly encompass physical activity and dietary optimization (Castrogiovanni et al., 2019; Szychlinska et al., 2019). Non-steroidal anti-inflammatory agents or the intra-articular administration of hyaluronic acid and corticosteroids constitute pharmacological interventions (Kloppenburg et al., 2018). Moreover, protracted usage of these pharmaceuticals can precipitate severe side effects, including gastrointestinal ulcers and perforations (Gregori et al., 2018; Chen et al., 2023). Furthermore, the recourse to artificial joint arthroplasty surgery may impose substantial trauma upon patients and is primarily indicated for those with advanced and severe manifestations of late-stage OA (Hou et al., 2021). Consequently, OA, a degenerative joint disorder characterized by pain and disability stemming from the deterioration of joint tissues, necessitates innovative approaches.

In contemporary times, regenerative therapy has emerged as a cutting-edge and swiftly advancing approach in the treatment of OA. The regenerative therapy is especially meaningful for joint preservation, which is critical for the demographic of relatively young, middle-aged patients (<65 years old) who maintain an active lifestyle (Hou et al., 2021). It serves to defer the inevitability of irreversible surgical interventions like joint arthroplasty, thereby averting the necessity for intricate and costly revision surgeries in later stages of life. For instance, platelet-rich plasma, derived from processed blood samples, furnishes crucial growth factors that hold the potential to facilitate the recovery process in OA (Paget et al., 2021). Furthermore, strategies centered around Mesenchymal Stem Cells (MSCs) have been extensively investigated for their capacity to self-renew, possess immunomodulatory properties, and exhibit the potential for multilineage differentiation (Yao et al., 2023). Additionally, MSC-derived exosomes have emerged as novel clinical biomarkers or therapeutic agents for individuals with OA. As an illustration, Tao et al (Wu et al., 2019). Employed a lentiviral system to overexpress miR-140-5p in synovial MSCs, resulting in consistently elevated levels of this miRNA in the exosomes released by these cells. When compared to unmodified exosomes, those abundant in miR-140-5p demonstrated an augmented capacity to induce chondrocyte proliferation and migration, effectively impeding cartilage degeneration in rat models of OA. Although the evidence supporting the impact of MSCs and exosomes on cartilage restoration is compelling, their durability and effectiveness warrant meticulous scrutiny to optimize their clinical potency. Additionally, alternative approaches within tissue engineering, such as hydrogels and scaffolds, have been deployed to enhance the precise delivery of therapeutic agents to specific cartilage defects (Wu et al., 2022).

In addition to regenerative therapies, joint distraction, a minimally invasive surgical procedure, entails delicately separating the two osseous extremities of a joint and maintaining them at a specific distance for a designated period through the use of an external fixation frame (Teunissen et al., 2022). First elucidated in the 1990 s, it has subsequently emerged as a pivotal joint-preserving modality for individuals afflicted with end-stage OA, who are candidates for joint replacement surgery (Aldegheri et al., 1994; Lafeber et al., 2006). Over the past 3 decades, joint distraction has exhibited substantial promise for integration into routine clinical practice.

This therapeutic approach has found clinical application in various joints affected by osteoarthritis, encompassing the knee, hip, ankle, and foot (Aldegheri et al., 1994; Van Valburg et al., 1995; Bain et al., 1998; Van Der Woude et al., 2017a). Among these, knee joint distraction (KJD) has garnered more comprehensive research attention. The inaugural trial of Knee Joint Distraction (KJD) was documented in 2007, featuring a retrospective examination of six Osteoarthritis (OA) patients who underwent a protocol combining hinged KJD employing a tailored frame over a span of 2-3 months, alongside bone marrow stimulation (Deie et al., 2007; Deie et al., 2010). Subsequently, in a 2017 Randomized Controlled Trial (RCT), 23 subjects received knee joint distraction while 46 subjects underwent High Tibial Osteotomy (HTO). The results unveiled a minimum increment of 0.8 ± 1.0 mm in Medial Compartment Joint Space Width (JSW) for the knee joint distraction cohort (*p* = 0.001), juxtaposed with 0.4 ± 0.5 mm for the HTO group (*p* < 0.001). This indicated a superior enhancement in minimum JSW for knee joint distraction (*p* = 0.05) (Van der Woude et al., 2017b). Concurrently, in the same year, a separate RCT was published, encompassing 20 OA patients treated with KJD and 40 patients subjected to Total Knee Arthroplasty (TKA) (Van der Woude et al., 2017c), This study also highlighted conspicuous clinical advancement and the reinstatement of tissue structure with KJD after 1 year, paralleling the progress observed in TKA recipients. Furthermore, a comprehensive 2-year follow-up investigation targeting severe knee osteoarthritis unveiled the sustained presence of both clinical and structural benefits subsequent to joint distraction. Remarkably, cartilage repair persisted, as corroborated by MRI imaging, and the newly generated tissue exhibited heightened mechanical resilience, exemplified by an augmented JSW under weight-bearing conditions (Wiegant et al., 2013). The prospective study conducted by Intema et al. reveals enduring outcomes. It demonstrates an initial augmentation in cartilage thickness compared to pre-treatment levels at the 1-year and 2-year evaluations. However, a gradual decline ensued thereafter. Nonetheless, at the 10-year assessment, cartilage thickness in both the tibia and femur still surpassed pre-treatment levels (Jansen et al., 2022). In a parallel study concentrating on severe ankle osteoarthritis, a substantial 73% of patients experienced and maintained clinical improvements, with an average duration of 10 years (Ploegmakers et al., 2005). These findings underscore the enduring protective effects of joint distraction on compromised joints.

An inquiry into the long-term ramifications and cost-effectiveness of joint distraction in knee osteoarthritis substantiated that the implementation of knee joint distraction (KJD) could lead to a reduction in the frequency of knee replacement procedures, particularly among younger age cohorts, inclusive of instances necessitating revision surgeries. At a willingness-to-pay threshold of 20,000 Euros per Quality-Adjusted Life Year (QALY) gained, the probability of commencing treatment with KJD being deemed cost-effective in comparison to initiating with total knee arthroplasty (TKA) exceeded 75% for all age strata, and exceeded an impressive 90%–95% for the younger age groups (van der Woude et al., 2016). These findings highlight the substantial cost-effectiveness and long-term benefits of joint distraction as a viable treatment option in the management of knee osteoarthritis.

A review has posited several potential mechanisms underlying joint repair and enhanced clinical outcomes. These encompass mechanical unloading and the maintenance of synovial fluid pressure oscillation, alongside periarticular bone alterations that impact stem cells and the overall joint milieu (Jansen and Mastbergen, 2022). In a pertinent research endeavor, the benefits of joint distraction were scrutinized in an osteoarthritis rat model, shedding light on the underlying mechanistic pathways. This investigation revealed a significant reduction in the serum IL-1β level within the joint distraction group. Moreover, there was a discernible attenuation of cartilage degeneration and abnormal subchondral bone changes, substantiated by lower histologic damage scores, a decreased percentage of MMP13 or Col X positive chondrocytes, diminished bone mineral density (BMD) and bone volume/total tissue volume (BV/TV), and a reduction in the number of Nestin or Osterix positive cells in the subchondral bone (Chen et al., 2015). In tandem with alterations in inflammation factors within the synovial fluid, the anabolic and catabolic processes underwent noteworthy transformations post KJD intervention. Teunissen et al. recently elucidated that in a canine model, proteoglycan and collagen type II content exhibited partial restoration, and there was a discernible augmentation in proteoglycan synthesis following a 10-week follow-up period subsequent to KJD treatment (Teunissen et al., 2023). Fascinatingly, the process of joint unloading through KJD leads to a persistent and substantial augmentation in both the size and density of SF-MSC colonies. The initial 3 weeks of joint distraction therapy were characterized by notable elevations in markers associated with MSC chondrogenic commitment, specifically gremlin 1 and growth differentiation factor 5 (GDF5). These markers are closely linked with the maintenance of a healthy cartilage homeostasis (Leijten et al., 2013; Kouroupis et al., 2019; Kania et al., 2020a; Kania et al., 2020b). All of these findings collectively suggest that joint distraction therapy encompasses a spectrum of therapeutic effects, underscoring the need for more extensive and in-depth investigations.

Despite the extensive body of research on joint distraction, a noticeable dearth exists in terms of comprehensive and meaningful analyses pertaining to publication trends in this domain. Hence, prior to embarking on further basic and clinical investigations, it is imperative to encapsulate the current focal points and frontiers within joint distraction for osteoarthritis treatment. Bibliometrics, leveraging quantitative analyses through mathematical and statistical methodologies to scrutinize published research outcomes, furnishes objective scientific metrics for researchers to monitor quantitative shifts, distributions, and patterns in the extant literature (Zhao et al., 2022). Presently, both the volume and caliber of research on joint distraction for osteoarthritis treatment remain uncharted territory. To address this void, a dedicated study is underway to encapsulate the present state of joint distraction in osteoarthritis treatment research, prognosticate prospective keywords and frontiers, and facilitate researchers in discerning the prevailing research trends and frontiers in this burgeoning field.

## 2 Materials and methods

### 2.1 Data acquisition and search strategies

**FIGURE 1:**
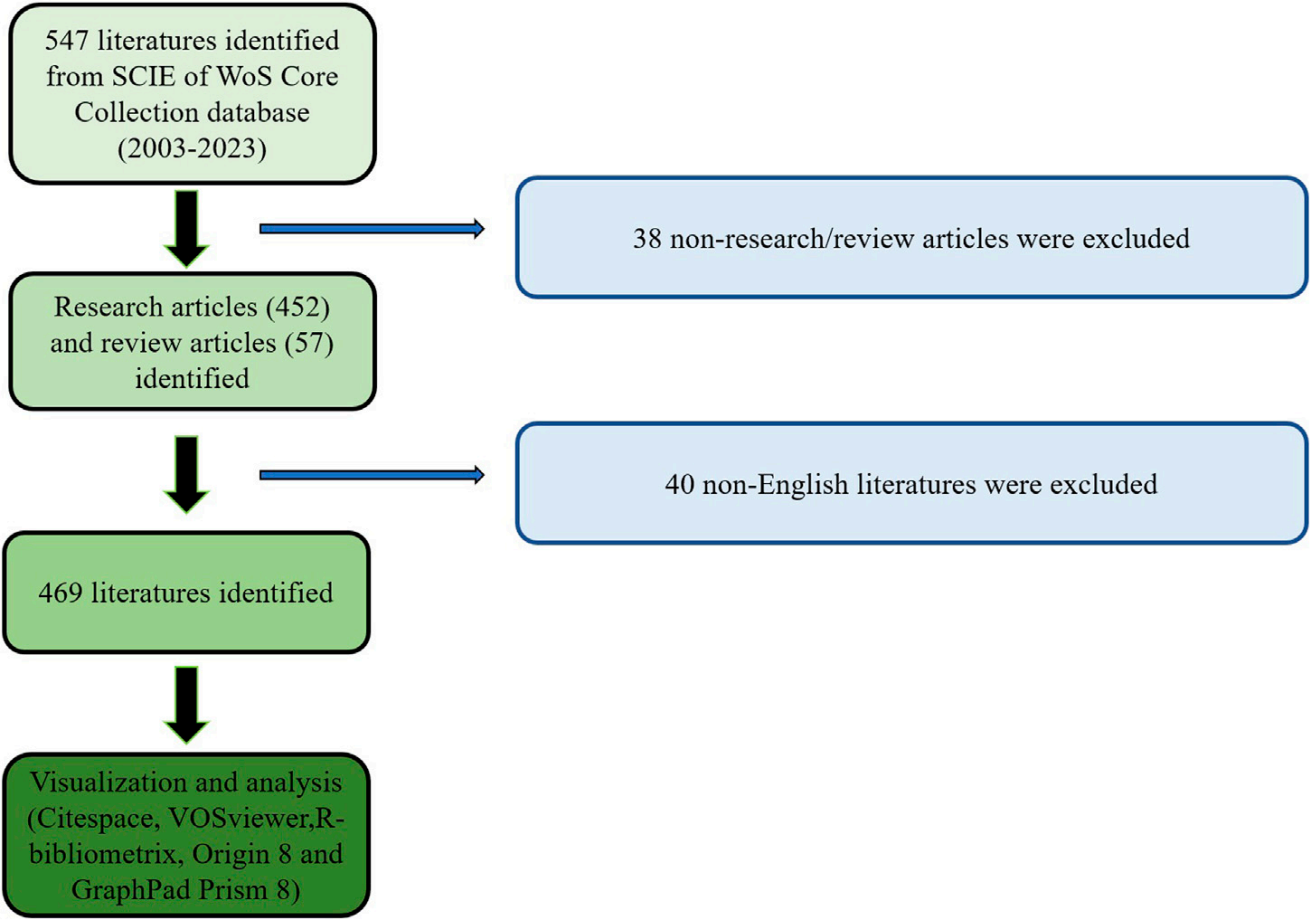
Flowchart depicting the article selection process.

The acquisition of publications related to research on joint distraction in osteoarthritis treatment was undertaken using the SCI-Expanded database within the Web of Science Core Collection (WoSCC) from Clarivate Analytics. Subsequently, studies pertinent to joint distraction in osteoarthritis treatment research were identified and subjected to bibliometric and visualized analyses, guided by established methodologies from previous studies (Ma et al., 2022; Zhang et al., 2022). The search parameters were set from 1 August 2003, to 1 August 2023, and the search formula was structured as follows: TS = (osteoarthritis OR degenerative arthritis) AND TS = (distraction OR distractor OR traction OR arthrodiastasis). Moreover, the publication criteria were delineated as follows: 1) The publications predominantly centered on the theme of joint distraction in osteoarthritis treatment; 2) Document types were restricted to Articles and Reviews. 3) Papers were required to be composed in English. The exclusion criteria were likewise specified as follows: 1) Themes were not aligned with joint distraction in osteoarthritis treatment; 2) Publications classified as meeting abstracts, proceedings papers, corrections, book chapters, letters, news, and the like were excluded (see Figure 1). A meticulous evaluation of these publications was conducted by two reviewers (LQP and RML), with any publications deemed irrelevant to the research topic of joint distraction in osteoarthritis treatment being manually filtered out. Additionally, consultations with experienced corresponding authors were undertaken to adjudicate on whether to incorporate any potentially relevant but initially excluded publications into the present study.

The basic information pertaining to the publications, encompassing details like journals, titles, authors, keywords, institutions, countries/regions, publication dates, as well as comprehensive statistics such as total citations, H-index, and average citation counts, was extracted by two authors (HL and RYZ) and subsequently imported into Excel 2021. Subsequently, bibliometric analyses and visualizations were conducted utilizing a suite of software applications, namely GraphPad Prism 8, Origin 2021, VOSviewer (Leiden University, Leiden, Netherlands, version 1.6.14) (Eck and Waltman, 2014) and CiteSpace (version 6.2.4) (Chen, 2016). These tools were instrumental in dissecting and visualizing the intricate landscape of publications related to joint distraction in osteoarthritis treatment, providing a comprehensive perspective on the scholarly contributions in this domain.

### 2.2 Bibliometric analysis and visualization

To start, annual publication trends and Relative Research Interest (RRI) were graphically represented using the curve-fitting function in GraphPad Prism 8. The RRI, calculated as the number of papers in a specific field divided by the total number of papers across all fields in a given year, provided insights into the field’s prominence relative to others. For the world map analysis, a methodology based on previous studies was employed (Xing et al., 2018). Additionally, the total publications from the top 10 countries between 2003 and 2023, along with a global trend prediction, were analyzed using Origin 2021 software. The Impact Factor (IF) of journals was obtained from the Journal Citation Reports for the year 2022.

Next, CiteSpace (version 6.2.4) software, developed by Professor Chen C, was employed for a comprehensive array of analyses including country/region and institution collaboration, dual-map overlay of journals, author collaboration, co-cited authors analysis, cluster detection of co-cited references and keywords, and scrutiny of references and keywords exhibiting pronounced citation bursts. The following parameters were configured in line with prior studies: link retaining factor (LRF = 3), look back years (LBY = 5), e for top N (e = 1), time span (2003–2023), years per slice (1), links (strength: cosine, scope: within slices), selection criteria (g-index: k = 25), and minimum duration (MD = 2 for keywords; MD = 5 for references) (Zhang et al., 2022). These parameters ensured a robust and accurate analysis of the extensive bibliographic data related to joint distraction in osteoarthritis treatment.

Furthermore, VOSviewer was employed in this study to build and visualize bibliometric networks, enabling the acquisition of more comprehensive information, including: 1) Co-citation analysis of journals and references. 2) Co-occurrence analysis of keywords. In the visual representation generated by VOSviewer, each node corresponds to an item, encompassing co-cited references and keywords. The size of the node corresponds to the number of publications associated with it, while the color denotes the respective publication year. The thickness of the lines connecting different nodes indicates the strength of collaboration or co-citation relationships, providing a visually intuitive representation of the intricate interconnections within the bibliographic data. This approach facilitates a deeper understanding of the thematic and conceptual linkages within the field of joint distraction in osteoarthritis treatment.

## 3 Results

### 3.1 Global contribution to the field

**FIGURE 2:**
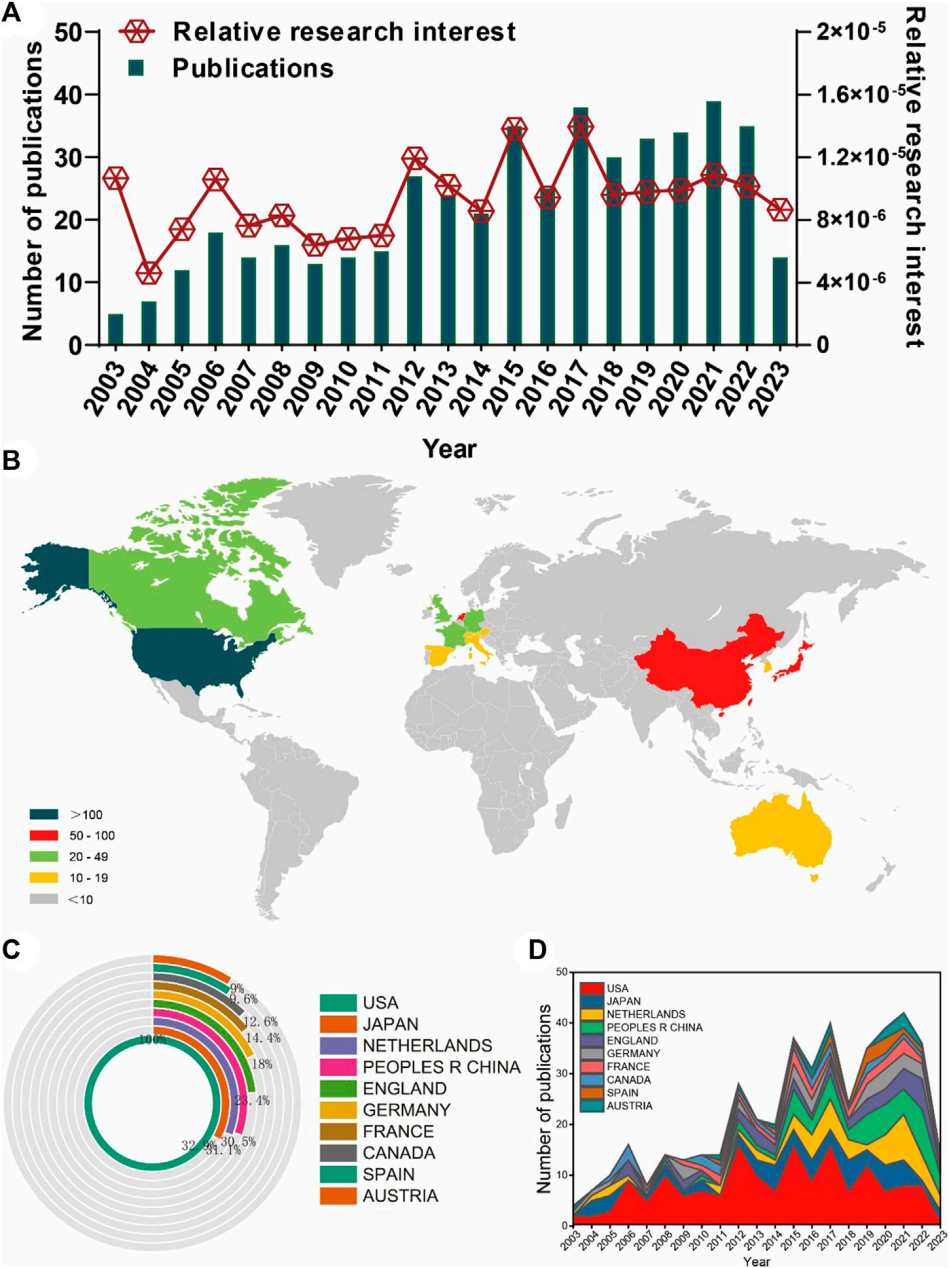
Global trends and countries/regions contributing to the research field regarding joint distraction in osteoarthritis treatment. **(A)** The annual number of publications related to joint distraction in osteoarthritis treatment. **(B)** A world map depicting distribution of joint distraction in osteoarthritis treatment. **(C)** The sum of joint distraction in osteoarthritis treatment related publications in the top 10 countries/regions. **(D)** The annual number of publications in the top 10 most productive countries from 2003 to 2023.

Based on the meticulously devised publication search strategy (as illustrated in Figure 1), a total of 469 publications conformed to the established criteria and were consequently incorporated into the final analysis. Over the period from 2003 to 2023, the annual publication count exhibited a gradual increment, characterized by fluctuations, surging from a modest five articles to an impressive tally of over thirty articles (as demonstrated in Figure 2A). Concurrently, the Relative Research Interest (RRI) demonstrated a relatively stable trend around a baseline level across the same period (as depicted in Figure 2A). In totality, contributions to the research domain of joint distraction in osteoarthritis treatment emanated from 38 distinct countries/regions. Notably, the United States took the lead, accounting for the lion’s share with 167 publications, constituting 35.61% of the total. This was followed by Japan (55 publications, 11.73%), the Netherlands (52 publications, 11.09%), China (51 publications, 10.87%), and England (39 publications, 8.32%) (as illustrated in Figures 2B, C, and summarized in Table 1). Furthermore, Figure 2D underscores the evolving landscape of publication contributions, with the United States having held the forefront position from 2003 to 2020. However, in recent years, other countries have made substantial strides, increasingly vying for the lead.

**TABLE 1:** The top 10 productive countries/regions related to joint distraction in osteoarthritis treatment.

Rank	Country/region	Article counts	Percentage (N/469)	Citation	Average citation	H index
1	United States	167	35.61	3,406	20.40	29
2	Japan	55	11.73	991	18.02	17
3	Netherlands	52	11.09	1,188	22.85	20
4	China	51	10.87	516	10.12	15
5	England	39	8.32	1,239	31.77	18
6	Germany	30	6.39	578	19.27	12
7	France	24	5.12	296	12.33	10
8	Canada	21	4.48	406	19.33	11
9	Spain	16	3.41	261	16.31	9
10	Austria	15	3.19	277	18.47	8

### 3.2 Distribution of countries/regions and institutions

**FIGURE 3:**
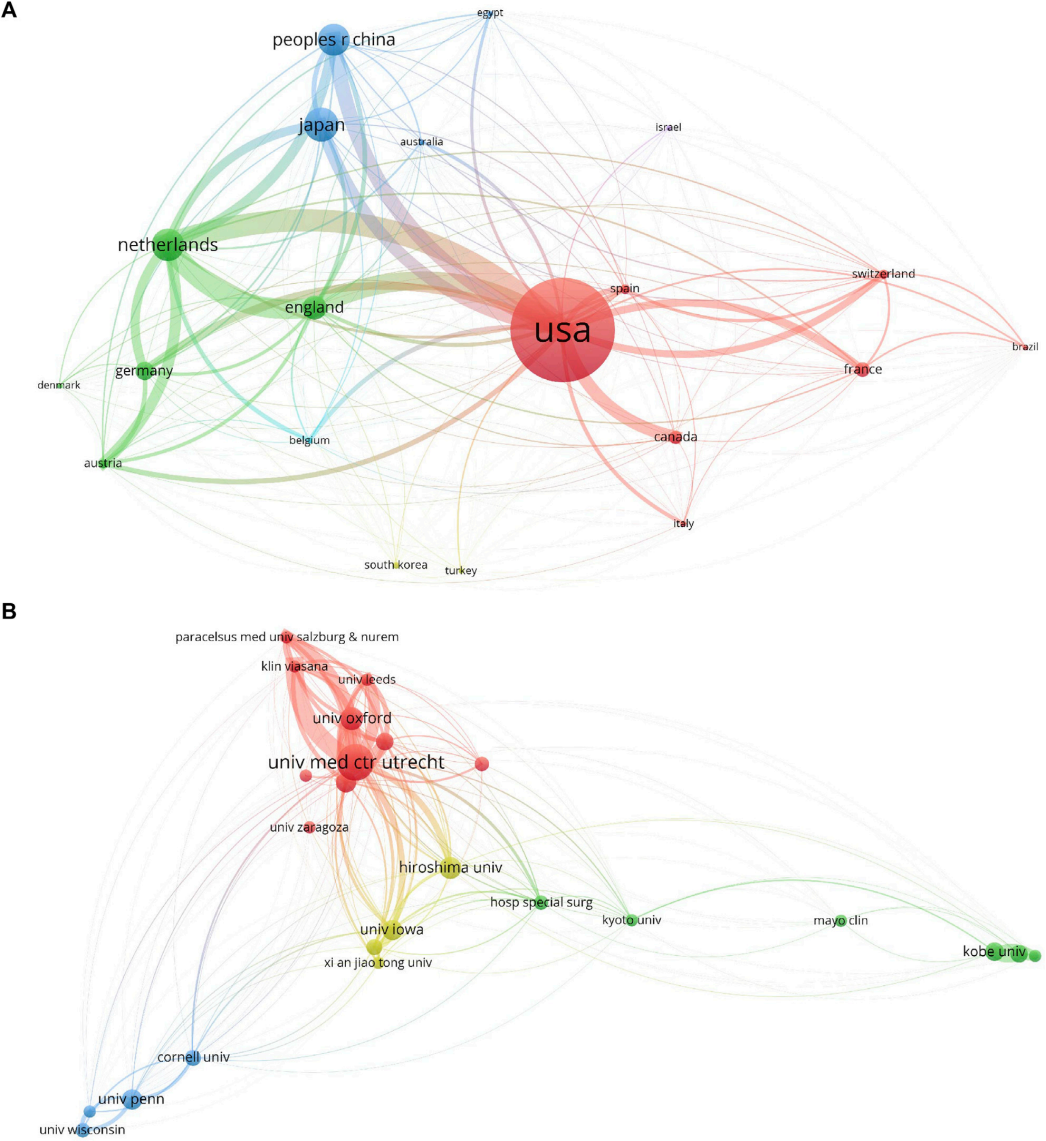
Mapping of countries/regions and institutions associated with joint distraction in osteoarthritis treatment. **(A)** Country/regional collaboration analysis. **(B)** Institutional collaboration analysis. The nodes represent countries/regions or institutions, and the lines connect them. The number of publications grows proportionally to the size of the nodes. The lines between the nodes represent the cooperation relationship, and the thickness of the connecting lines represents the strength of their cooperation, the closer the cooperation, the thicker the connecting lines.

All 469 publications emanated from a total of 38 different countries and involved 781 distinct institutions. Notably, the top 10 countries/regions exhibited a global dispersion, with representation from North America, Asia, and Western Europe (as detailed in Table 1). Among these, the United States stood out significantly, contributing to over a third of the total publications, a substantial lead over other countries/regions. Furthermore, Table 1 outlines that the United States also boasted the highest total citations (3,406) and an impressive H-index of 29, surpassing all other countries. England exhibited the highest average citation rate (31.77), closely followed by the Netherlands (22.85) and the United States (20.40), underscoring their pivotal positions in terms of published papers. These three countries together accounted for over 55% of the total publications, emphasizing their dominant influence in the field. Subsequently, the collaborative landscape among countries/regions was visually represented in Figure 3A. In this depiction, the size of each node corresponds to the number of documents, with the United States displaying the most robust collaboration strength.

Moving to Table 2, it is evident that the top 10 most productive institutions were based in the United States, England, Japan, and the Netherlands. Notably, the University of Iowa, despite not having the largest total publication count, exhibited substantially higher average citations compared to other institutions. Among these, Utrecht University led with 40 papers and 1,024 citations, followed by Utrecht University Medical Center (38 papers, 932 citations) and the University of Oxford (13 papers, 442 citations). Of the top 10 productive institutions, the University of Iowa displayed the highest average citation rate (41.27), followed by Utrecht University (25.6), and Utrecht University Medical Center (24.53). Furthermore, the institutional cooperation analysis, depicted in Figure 3B, unveiled Utrecht University, Utrecht University Medical Center, and the University of Oxford as the leading institutions in terms of collaborations with other research entities.

**TABLE 2:** The top 10 institutions published literature related to joint distraction in osteoarthritis treatment.

Rank	Institution	Country	Article counts	Percentage (N/469)	Total citations	Average citation
1	Utrecht University	Netherlands	40	8.529	1,024	25.6
2	Utrecht University Medical Center	Netherlands	38	8.102	932	24.53
3	University Of Oxford	England	13	2.772	442	34
4	HIROSHIMA UNIVERSITY	Japan	11	2.345	144	13.09
5	University Of California System	United States	11	2.345	223	20.27
6	University Of Iowa	United States	11	2.345	454	41.27
7	University Of Pennsylvania	United States	11	2.345	187	17
8	Kobe University	Japan	9	1.919	146	16.22
9	Sint Maartens Clinic	Netherlands	9	1.919	234	26
10	Udice French Research Universities	France	8	1.706	95	11.88

### 3.3 Analysis of journals and research areas

**FIGURE 4:**
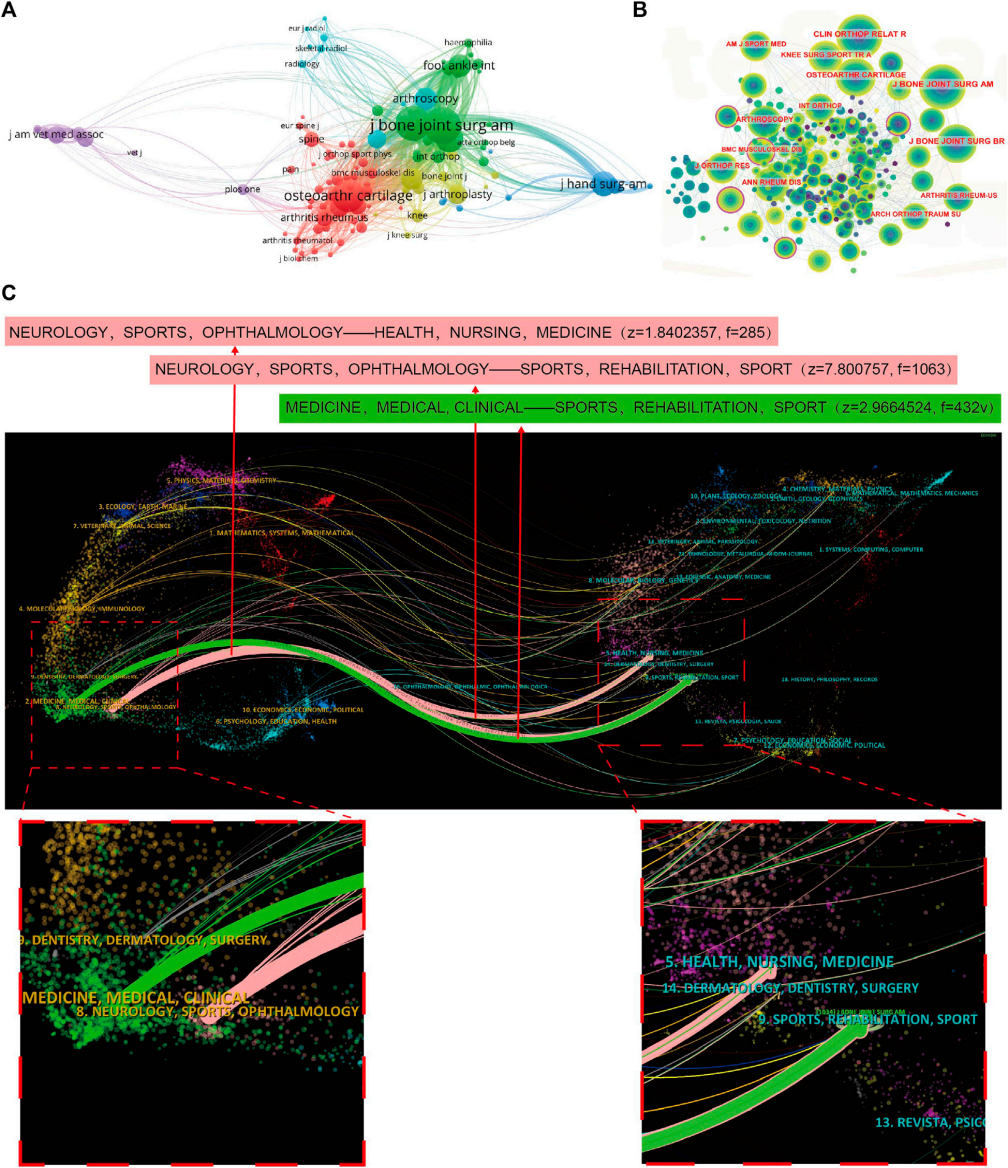
Articles published in different journals on joint distraction in osteoarthritis treatment. **(A)** Network map of journals that were co-cited in more than 20 citations. **(B)** Journals collaboration analysis. **(C)** The dual-map overlay of journals related to joint distraction in osteoarthritis treatment.

Between 2003 and 2023, a total of 469 articles were published across 183 journals. The top 10 journals with the highest number of publications are listed in Table 3, along with their most recent Impact Factors (IF). “Osteoarthritis And Cartilage” led the pack with 21 publications, constituting 4.478% of all articles, followed by “Journal of Hand Surgery American Volume” (15, 3.198%), “Journal of Foot Ankle Surgery” (13, 2.772%), “Foot Ankle International” (12, 2.559%), and “Knee Surgery Sports Traumatology Arthroscopy” (12, 2.559%). Notably, among these top 10 journals, “Osteoarthritis And Cartilage” boasted the highest IF of 7.0, followed by “Knee Surgery Sports Traumatology Arthroscopy” (3.8) and “Foot and Ankle International” (2.7). Additionally, an analysis of journals that were co-cited more than 20 times revealed the top 5 journals with the highest total link strength: “Journal of Bone and Joint Surgery- American Volume” (total link strength: 37,554), “Clinical Orthopaedics and Related Research” (total link strength: 34,373), “Osteoarthritis And Cartilage” (total link strength: 33,053), “Journal of Bone and Joint Surgery-British Volume” (total link strength: 23,312), and “Foot Ankle International” (total link strength: 17,923) (as depicted in Figure 4A). In Figure 4B, we can see that the closely related journals are concentrated in the fields of orthopedics, sports medicine, and rheumatology.

**TABLE 3:** The top 10 productive journals related to joint distraction in osteoarthritis treatment.

Rank	Journal	Article counts	Percentage (N/469)	Citation per article	If (2022)
1	Osteoarthritis And Cartilage	21	4.478	32.95	7
2	Journal Of Hand Surgery American Volume	15	3.198	32.53	1.9
3	Journal Of Foot Ankle Surgery	13	2.772	7.62	1.3
4	Foot Ankle International	12	2.559	19.33	2.7
5	Knee Surgery Sports Traumatology Arthroscopy	12	2.559	19.67	3.8
6	Orthopaedics Traumatology Surgery Research	12	2.559	12.75	2.3
7	Archives Of Orthopaedic And Trauma Surgery	11	2.345	31	2.3
8	Knee	10	2.132	14	1.9
9	American Journal Of Veterinary Research	8	1.706	19	1
10	Clinics In Podiatric Medicine And Surgery	8	1.706	4.88	0.6

The identified publications were further categorized into 47 distinct research areas. Among the top 10 well-represented research areas, Orthopedics accounted for the majority with 274 records, constituting 58.42% of all articles, followed by Surgery (134, 28.57%), and Sport Sciences (54, 11.51%) (as outlined in Table 4). Additionally, a dual-map overlay of journals was conducted to explore the citation relationships between cited and citing journals, revealing three primary citation paths marked in pink and green (Zhang et al., 2022). One of the pink paths indicated that papers in the field of health/nursing/medicine were predominantly cited by papers in neurology/sports/ophthalmology, while the other demonstrated that documents in sports/rehabilitation/sport were mainly cited by neurology/sports/ophthalmology. The orange path indicated that publications in the area of sports/rehabilitation/sport were cited by articles in the area of medicine/medical/clinical. Enlarged figures in Figure 4C provide a detailed view of the distinct citing trajectories of both citing and cited journals.

**TABLE 4:** The top 10 well-represented research areas related to joint distraction in osteoarthritis treatment.

Rank	Research areas	Records	Percentage (N/469)	Total citations
1	Orthopedics	274	58.42	5,288
2	Surgery	134	28.57	2,815
3	Sport Sciences	54	11.51	1,262
4	Veterinary Sciences	45	9.59	556
5	Rheumatology	40	8.53	1,366
6	General Internal Medicine	25	5.33	379
7	Rehabilitation	20	4.26	283
8	Engineering	19	4.05	197
9	Neurosciences Neurology	10	2.13	278
10	Research Experimental Medicine	10	2.13	105

### 3.4 Authors analysis

**FIGURE 5:**
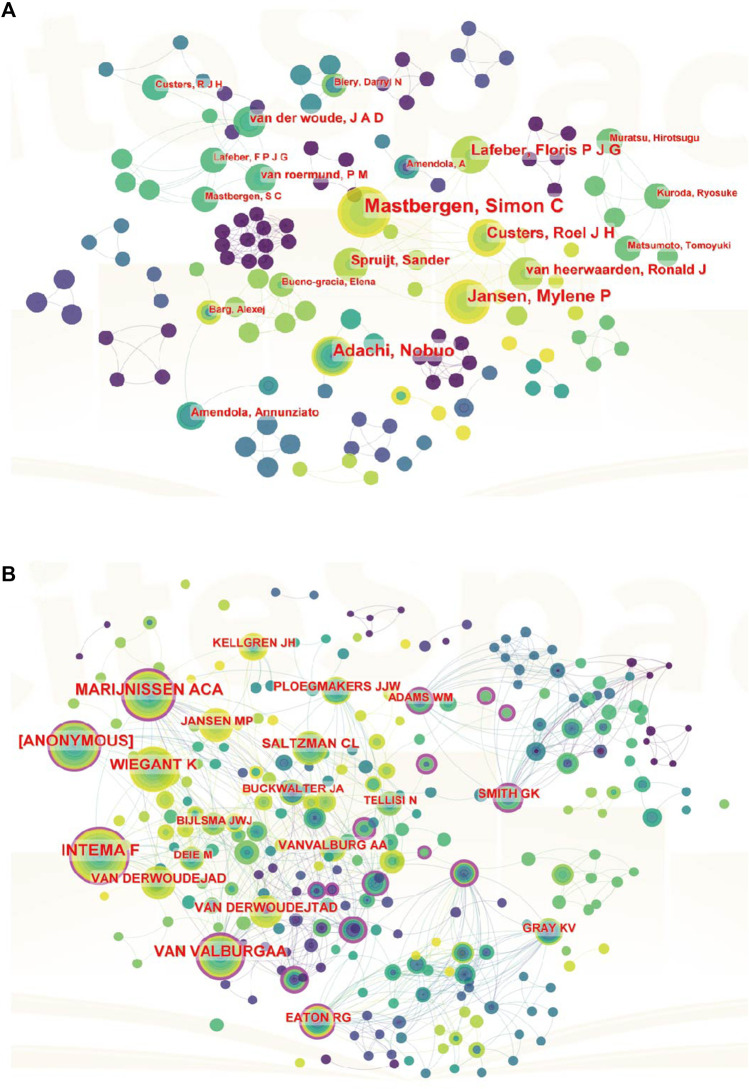
A CiteSpace network visualization of author collaboration analysis and co-cited authors regarding joint distraction in osteoarthritis treatment. **(A)** Author collaboration analysis. **(B)** Network visualization diagram of the co-cited authors of the publications. Author timeline visualization from 2003 to 2023. Author collaboration or co-cited authors are indicated by the node. The co-citation relationship is indicated by the line connecting the nodes. The node area grows as the number of co-citations increases. The colors represent different years, the color changes from purple to yellow from 2003 to 2023.

The top 10 authors who made significant contributions to the field of joint distraction for the treatment of osteoarthritis, in terms of both publications and citations, are presented in Table 5. Mastbergen SC emerged as the leading contributor with 29 publications, closely followed by Lafeber FPJG (26 publications), and Jansen MP (15 publications). Additionally, among these prolific authors, Lafeber FPJG garnered the highest total citations, amassing 974, followed by Mastbergen SC (784 citations) and Van Roermund PM (578 citations). Moreover, an analysis of author cooperation was conducted to visually represent the collaborative relationships among researchers. Additionally, a co-cited author network visualization diagram was established (Figures 5A, B). In Figure 5, the size of nodes corresponds to the number of co-citations, while the color signifies the publication year. Noteworthy co-cited authors include Smith GK (159 Citations), Marijnissen ACA (104 Citations), Van Valburg AA (102 Citations), Intema F (97 Citations), and Jansen MP (97 Citations), underscoring their significant influence and recognition within the field.

**TABLE 5:** The top 10 authors with the most publications and citations on joint distraction in osteoarthritis treatment.

Rank	High published authors	Article counts	Article counts (N/469)	Total citations	H-index
1	Mastbergen SC	29	6.183	784	16
2	Lafeber FPJG	26	5.544	974	17
3	Jansen MP	15	3.198	187	8
4	Custers RJH	14	2.985	307	10
5	Van Roermund PM	14	2.985	578	11
6	Adachi N	11	2.345	144	6
7	Van Heerwaarden RJ	11	2.345	223	8
8	Spruijt S	10	2.132	209	7
9	Kuroda R	8	1.706	146	6
10	Matsumoto T	8	1.706	146	6

### 3.5 Citation and co-citation analysis of reference

**FIGURE 6:**
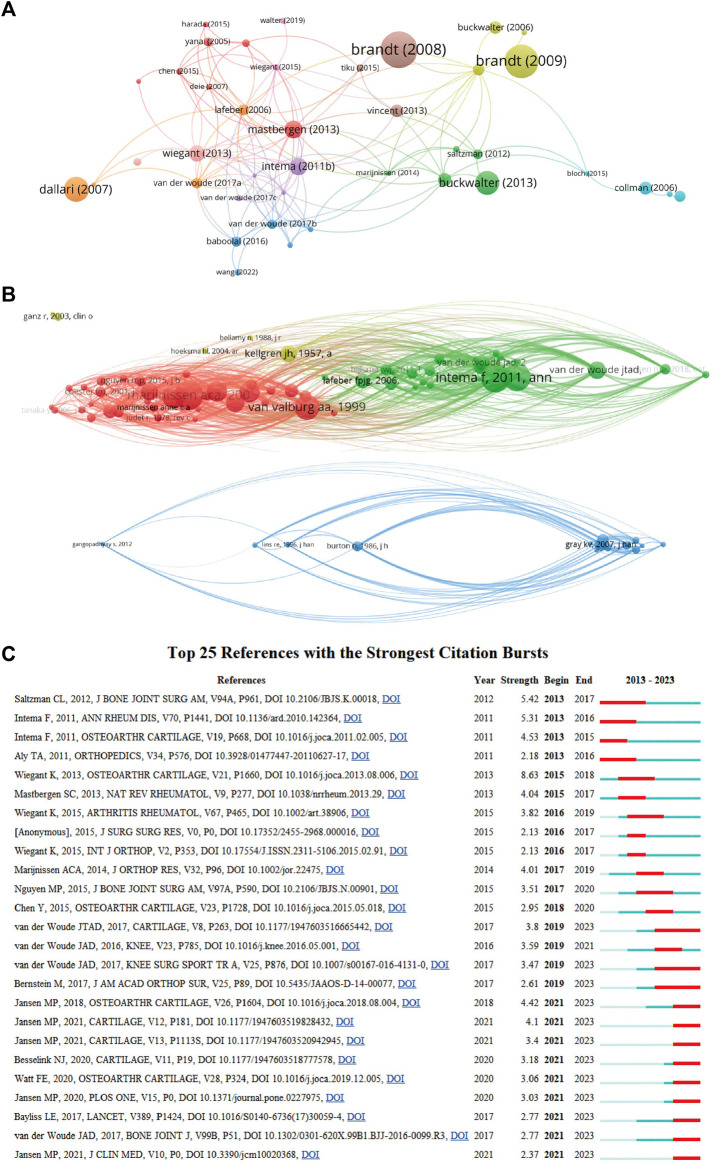
Mapping of documents and references in studies on joint distraction in osteoarthritis treatment. **(A)** Network map of citation analysis of documents with more than 25 citations. **(B)** Network map of co-citation analysis of references based on CiteSpace. **(C)** Top 25 references with strongest citation bursts of publications related to joint distraction in osteoarthritis treatment.

A thorough analysis of documents in this field with over 25 citations was conducted, totaling 106 papers, visualized using VOSviewer (as illustrated in Figure 6A). The top 5 most cited publications are also outlined. In detail, “Which physical examination tests provide clinicians with the most value when examining the shoulder? Update of a systematic review with meta-analysis of individual tests” garnered the highest number of citations, accumulating 254 in total. Following closely, “Effect of high tibial flexion osteotomy on cartilage pressure and joint kinematics: A biomechanical study in human cadaveric knees: Winner of the AGA-DonJoy Award 2004" secured the second position with 221 citations. The third most cited paper was “Etiopathogenesis of osteoarthritis,” with 211 citations. Furthermore, the co-cited references were visualized using VOSviewer (as depicted in Figure 6B). From this analysis, the top 5 references with the highest number of citations were identified. Notably, Intema F (2011; 53 citations) and Marijnissen ACA (2002; 53 citations) shared the top spot, followed by Wiegant K (2013; 50 citations), Van Valburg AA (1999; 45 citations), and Ploegmakers JJW (2005; 42 citations).

Moreover, references with citation bursts serve as valuable indicators of literature frequently cited in a specific domain over a period of time (Ma et al., 2022). I In this study, the top 25 references exhibiting the strongest citation bursts were presented in Figure 6C, along with the corresponding duration of the burst. Notably, the article titled “Motion Versus Fixed Distraction of the Joint in the Treatment of Ankle Osteoarthritis,” published in 2012, held the top position with a strength of 5.42. Additionally, the citation bursts of articles authored by JTAD van der Woude persisted the longest, spanning from 2019 to 2023. This insightful analysis highlights the enduring impact and influence of specific references within the domain of joint distraction for the treatment of osteoarthritis.

### 3.6 Co-occurrence analysis of keywords

**FIGURE 7:**
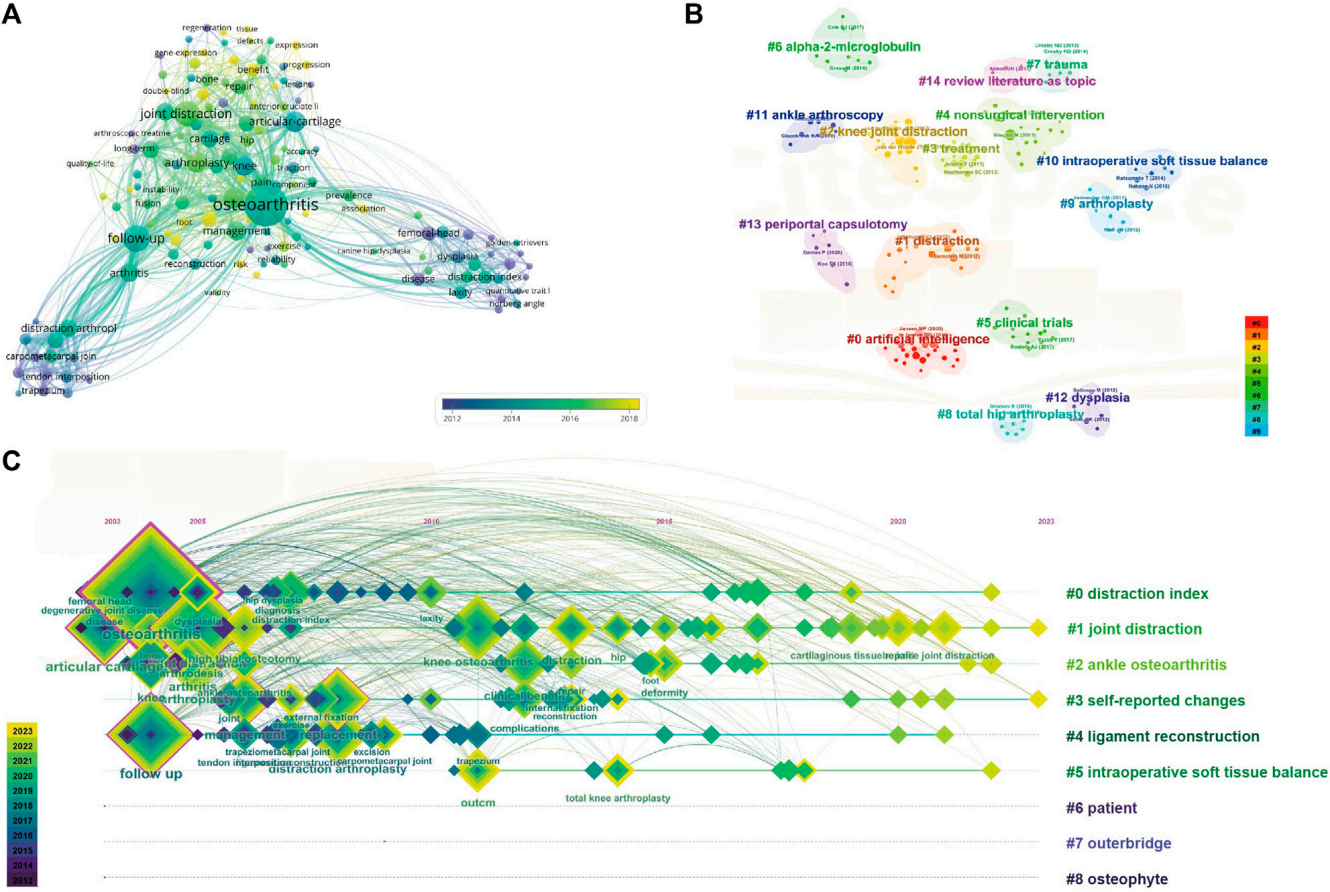
Mapping of keywords in studies on joint distraction in osteoarthritis treatment. **(A)** Distribution of keywords according to average publication year (Purple: earlier, yellow: later) by Vos Viewer. **(B)** Network visualization of keywords by Citespace. **(C)** Keyword timeline visualization from 2003 to 2023 by Citespace.

The co-occurrence cluster analysis of keywords was performed using both CiteSpace and VOSviewer to capture research frontiers in the field. Initially, a network map was constructed by VOSviewer to analyze the distribution of keywords based on their average publication year (with dark blue denoting earlier and yellow indicating later) (as displayed in Figure 7A). In total, 145 keywords were identified, with the five most frequently occurring being: osteoarthritis (total link strength: 607), follow-up (total link strength: 302), joint distraction (total link strength: 228), distraction arthroplasty (total link strength: 172), and articular cartilage (total link strength: 162). The majority of keywords were published prior to 2019, while terms such as high tibia osteotomy, mesenchymal stem cells, and supramalleolar osteotomy emerged as relatively new keywords after 2020. Subsequently, the clusters were categorized into 14 distinct aspects, as depicted in Figure 7B: artificial intelligence (cluster 0), distraction(cluster 1), knee joint distraction (cluster 2), treatment (cluster 3), nonsurgical intervention (cluster 4), clinical trials (cluster 5), alpha-2-microglobulin (cluster 6), trauma (cluster 7), total hip arthroplasty (cluster 8), arthroplasty (cluster 9), intraoperative soft tissue balance (cluster 10), ankle arthroscopy (cluster 11), dysplasia (cluster 12) and periportal capsulotomy (cluster 13).

Moreover, Figure 7C illustrates the time dynamic evolution of keyword clusters, as delineated by CiteSpace. In total, nine clusters were identified: distraction index (cluster 0), joint distraction (cluster 1), ankle osteoarthritis (cluster 2), self-reported changes (cluster 3), ligament reconstruction (cluster 4), intraoperative soft tissue balance (cluster 5), patient (cluster 6), and outerbridge (cluster 7), and osteophyte (cluster 8), among which cluster 0, cluster 1, cluster 2, cluster 3, cluster 4, cluster 6 and cluster 7 were former appeared hotspots, cluster 5 was the mid-period appeared hotspot, and all clusters except for cluster 6, 7, 8 are present research hotspots.

**FIGURE 8:**
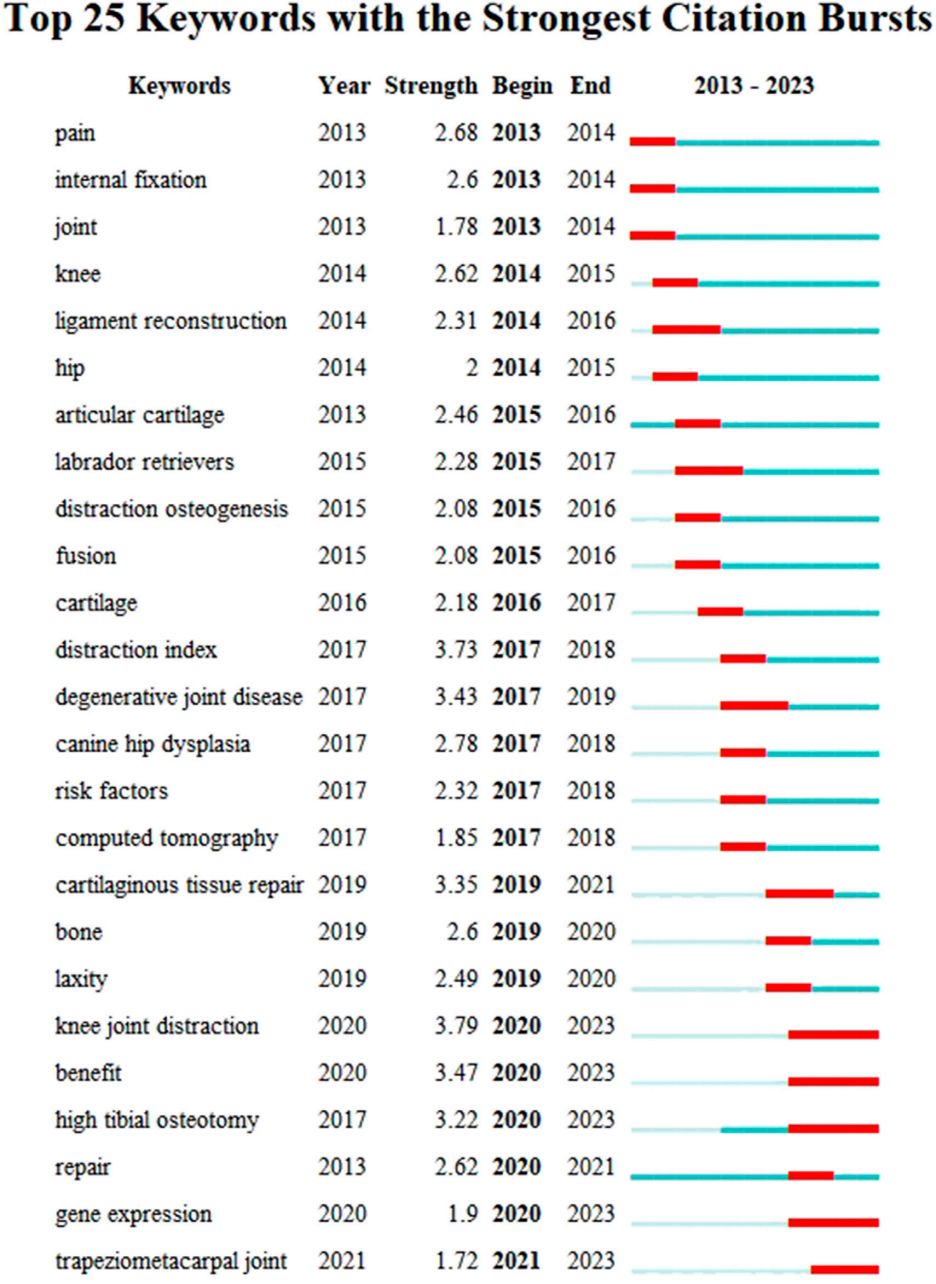
Top 25 keywords with the strongest citation bursts of publications related to joint distraction in osteoarthritis treatment.

Furthermore, CiteSpace’s algorithm was employed to scrutinize the burst of keywords, leading to the identification of the top 25 keywords with the most pronounced citation bursts (as shown in Figure 8). Notably, “pain” exhibited the strongest citation burst with a strength of 2.68, followed by “internal fixation” (strength = 2.6) and “joint” (strength = 1.78). Keywords such as “knee joint distraction,” “benefit,” “high tibial osteotomy,” and “gene expression” displayed the longest burst durations, each spanning 4 years from 2020 to 2023. Additionally, keywords such as “ligament reconstruction” (2014–2016), “labrador retrievers” (2015–2017), “degenerative joint disease” (2017–2019), “cartilaginous tissue repair” (2019–2021), and “trapeziometacarpal joint” (2021–2023) also exhibited notable burst periods. Interestingly, “knee joint distraction,” “high tibial osteotomy,” “gene expression,” and “trapeziometacarpal joint” were the keywords with the most recent outbreak of citations, indicating that research in these areas may represent future research hotspots.

## 4 Discussion

In recent decades, joint distraction has garnered global research attention and demonstrated its efficacy as a potent modality in the treatment of osteoarthritis. Significantly, Joint Distraction Technology confers several advantages over conventional joint-preserving interventions for end-stage osteoarthritis, exhibiting a burgeoning potential for integration into routine clinical practice. Nevertheless, a pivotal challenge in joint distraction research lies in the dearth of bibliometric analyses, impeding the identification of the domain’s knowledge framework, evolution, and focal research areas. Therefore, this study undertakes the inaugural bibliometric analysis of literature spanning from 2003 to 2023, leveraging CiteSpace and VOSviewer, to delineate the contemporary research landscape and prognosticate forthcoming focal points in joint distraction within the realm of osteoarthritis research.

### 4.1 Trends of joint distraction in osteoarthritis research

Our study reveals a notable increase in publications per annum from 1 August 2003 to 1 August 2022. Furthermore, a modest upturn in the Relative Research Interest (RRI) was observed in recent years. Additionally, approximately 40 countries have contributed to relevant studies in this domain. Notably, the United States exhibited the highest publication output (167, 35.61%). Table 1 demonstrates that the United States also led in total citations and possessed the highest H-index, underscoring its status as a prolific and pivotal contributor in this field. England exhibited the highest average citation rate, indicative of the exceptional quality of its contributions. Intriguingly, the Netherlands and the United States ranked second and third, respectively, in terms of average citations. Within the realm of scientific institutions, Utrecht University, University of Oxford, and Hiroshima University played active roles in advancing the research frontier. Remarkably, the top 10 institutes were exclusively situated in developed nations. Hence, this collective evidence suggests that a surge of studies characterized by profound insights and comprehensive knowledge on joint distraction in osteoarthritis research is anticipated in the forthcoming years.

### 4.2 Quality and status of global publications

In addition to the institutional analysis, we delved into the journals associated with publications, and the findings are delineated in Table 3. Notably, the journals “Osteoarthritis And Cartilage,” “Journal of Hand Surgery American Volume,” and “Journal of Foot Ankle Surgery” emerged as the most prolific publishers. In terms of Impact Factor (IF), the journals “Osteoarthritis And Cartilage,” “Knee Surgery Sports Traumatology Arthroscopy,” and “Foot and Ankle International” exhibited the highest IF values. It is foreseeable that, considering both quantity and quality, the top 10 listed journals are poised to serve as prime outlets for forthcoming high-caliber research. Furthermore, a co-citation analysis predicated on journals was executed to gauge the influence of publications, quantifying the total citation count. Figure 4A illustrates that the “Journal of Bone and Joint Surgery-American Volume” made the most remarkable contributions in this domain. Among the top 10 research orientations, eight are rooted in clinical studies, while two emanate from biological research, indicative of the pervasive interdisciplinary synergy within this domain. The dual-map analysis also mirrors the research focal points in health, sports, rehabilitation, and clinical studies.

Turning our attention to authors, we present the most prolific authors in Table 5. The top-ranking authors, who have authored a substantial volume of studies, are seasoned veterans who enjoy the highest repute, and are anticipated to continue driving advancements in joint distraction in osteoarthritis research. Additionally, the collaboration analysis depicted in Figure 5A reveals that collaborative efforts among authors within the same country are relatively frequent, signaling the need for increased international academic connectivity and communication. As portrayed in Figure 5B, Marijnissen ACA, Van Valburg AA, Wiegant K, Intema F, and Smith GK emerge as the preeminent authors with the highest co-citation frequency, exemplifying the international acclaim and recognition garnered by these researchers in this field.

The impact of published literature was assessed through citation analysis of documents (Figure 6A) and co-citation network analysis (Figure 6B). The most cited article was a systematic review elucidating the paramount value of physical examination tests for clinicians examining the shoulder, furnishing crucial diagnostic insights for shoulder injuries (Hegedus et al., 2012). Another seminal study by JD Agneskirchne et al. scrutinized the effects of high tibial flexion osteotomy on joint kinematics (Agneskirchner et al., 2004). Among the five most cited articles, a predominant share belong to clinically-oriented genres, focusing on clinical treatment, systematic review, and experimental clinical studies in sports medicine. Additionally, in the co-citation analysis of references, the pivotal publication authored by Intema F et al. warrants noteworthy attention.

### 4.3 Research hotspots and frontiers

The co-occurrence analysis of keywords, along with burst analysis, provides valuable insights into the prevailing trends and emerging focal points within the realm of joint distraction in osteoarthritis research. As depicted in Figure 8, the keyword “pain” exhibits the highest frequency of citation outbreaks, indicative of its foundational significance in this research domain. Figure 7A highlights prominent research clusters encompassing key terms such as “osteoarthritis,” “joint distraction,” “articular cartilage,” “distraction index,” and “follow-up.” Furthermore, Figure 8 illustrates that joint distraction in osteoarthritis spans a diverse array of research areas. These publications, to a certain extent, serve as a barometer for the contemporary trajectories and frontiers within this field. The co-occurrence network of keywords was constructed based on the identification of keywords in the titles and abstracts of all incorporated publications. In essence, we have categorized our findings into four principal sections: Novel joint distraction techniques, distraction arthroplasty, joint repair mechanisms, and combinational surgical treatments. These results not only align with promising hotspots in the domain of joint distraction in osteoarthritis research but also furnish projections for the prospective avenues of inquiry in this field.

#### 4.3.1 Novel joint distraction techniques

The co-occurrence analysis of keywords has brought to light three pivotal areas warranting further attention: “joint distraction,” “distraction index,” and “artificial intelligence.” The innovation in joint distraction devices has emerged as an alluring focal point, as these devices afford the advantage of maintaining continuous distraction tension without the risk of loosening. Notably, cutting-edge devices now permit lower leg rotation and near-complete range of motion for the knee joint. For instance, Kamei et al. have pioneered the development of novel joint distraction devices utilizing magnetic force, resulting in a discernible increase in lateral joint space. Notably, this increase demonstrated remarkable stability between post-distraction and post-weight-bearing phases (Kamei et al., 2013). Furthermore, artificial intelligence has found application in the analysis of radiographic changes. A study by Jansen et al. optimized automatic radiographic measurement methodologies, subsequently establishing a correlation between clinical and structural improvements, changes in SF biomarker levels, and even alleviation of pain experienced by these patients (Jansen et al., 2023).

#### 4.3.2 Distraction arthroplasty

A pivotal facet of joint distraction research lies in its clinical applications for osteoarthritis treatment. Our co-occurrence analysis of keywords reveals pertinent terms such as “ankle arthroscopy,” “trauma,” and “total hip arthroplasty.” Ankle osteoarthritis, being a complex condition, often necessitates end-stage treatments such as arthrodesis or arthroplasty. Surgeries like supramalleolar osteotomy, ankle arthrodesis, and total ankle arthroplasty have been explored as potential interventions for ankle osteoarthritis (Arshad et al., 2022). In examining the early functional outcomes of ankle distraction arthroplasty, Zhao et al. demonstrated benefits in promoting cartilage regrowth and reducing subchondral bone density. However, it was also observed that there is a relatively high failure rate, particularly among obese patients and those with large talar tilt angles (Zhao et al., 2017). Furthermore, developmental dysplasia of the hip, a common precursor to secondary hip osteoarthritis, may ultimately necessitate total hip arthroplasty (THA) at a younger age (Bicanic et al., 2014). Joint distraction presents itself as another potential therapeutic avenue in such cases. For instance, long-axis distraction mobilization (LADM), a manual therapy modality with varying dosages in terms of force, amplitude, rate, repetition, and duration, finds extensive application in orthopedic treatment. (Snodg et al., 2006; Estébanez-de-Miguel et al., 2018). Nonetheless, it is crucial to calibrate forces appropriately, avoiding extremes that could place undue stress on anatomical structures, potentially leading to clinical trial failures (Jull and Moore, 2002). Notably, high-grade mobilization techniques appear to be more efficacious in enhancing joint mobility and diminishing disability. A study confirmed that high-force LADM notably increased hip range of motion in patients with hip osteoarthritis (Estébanez-de-Miguel et al., 2018). These innovative applications of joint distraction hold promise for addressing osteoarthritis across diverse anatomical sites. The specific intensity of mobilization force emerges as a potential therapeutic target in osteoarthritis treatment, warranting further in-depth exploration in the future.

#### 4.3.3 Joint repair mechanisms

There is a pressing need for innovative regenerative mechanisms in joint distraction, as various distraction treatments entail the maintenance of synovial fluid pressure oscillation, periarticular bone alterations, and the presence of stem cells and a conducive joint milieu for cartilage regeneration (Jansen and Mastbergen, 2022). Given the fundamental role of mechanical stress in cartilage regeneration and repair, it is imperative to thoroughly investigate the effects induced by joint distraction in order to elucidate and potentially expedite the process of cartilage regeneration. For instance, it has been observed that at higher nominal strain magnitudes (50%–70%), mechanical compression can lead to joint tissue injury (Kurz et al., 2001), ultimately culminating in chondrocyte death via necrosis and apoptosis at the highest strain magnitudes (70%–90%) (Natoli et al., 2008; Stolberg‐Stolberg et al., 2013). These findings suggest that interventions aimed at temporarily reducing strain magnitude and shear stress could create a more favorable mechanical environment conducive to repair. Additionally, delving into the underlying reparative functions of endogenous subchondral bone mesenchymal stem cells (SB-MSCs) and synovial fluid-derived MSCs (SF-MSCs) represents a crucial research avenue for advancing joint distraction treatment. Notably, joint unloading through Knee Joint Distraction (KJD) yields a sustained and substantial augmentation in the size and density of SF-MSC colonies. Furthermore, there is an increase in the expression of the pivotal cartilage core protein, aggrecan, coupled with a decrease in the pro-inflammatory chemokine CCL2 (also known as MCP1) during joint distraction (Sanjurjo-Rodriguez et al., 2020). Therefore, temporary unloading leads to transcriptional changes in SF-MSCs that might favor the advances in cartilage regeneration and repair.

#### 4.3.4 Joint diseases adjuvant applications

While various distraction devices, such as custom articulated distraction device (Deie et al., 2007), Ilizarov circular frame (Deie et al., 2007), Monotube^®^ Triax™ external fixation system (Van der Woude et al., 2017a), and KneeReviver frame (Jansen et al., 2020), have demonstrated efficacy in osteoarthritis treatment, joint distraction is also applied in some joint diseases. These include intraoperative soft tissue balancing, ligament reconstruction, and osteotomy. Consequently, there is a pressing need to expand advanced therapeutic interventions, catering not only to joint injuries but also to cartilage regeneration. In the context of anterior cruciate ligament (ACL) reconstruction, joint distraction therapy has been applied to monitor structural changes throughout the healing process of bone-tendon-bone reconstruction. Research conducted by Bedi et al. revealed that a delayed application of cyclic axial load post anterior cruciate ligament reconstruction led to enhanced mechanical and biological parameters in tendon-to-bone healing, in comparison to immediate loading or prolonged postoperative knee immobilization (Bedi et al., 2010). Moreover, in the realm of total knee arthroplasty, it has been established that a greater joint distraction force corresponds to a larger varus ligament balance and joint gap (Nagai et al., 2014). Collectively, these studies underscore the imperative to investigate the regenerative potential and clinical advantages of joint distraction treatment for more specific joint pathologies.

### 4.4 Strengths and limitations

While this study provides a valuable overview and research guidance for joint distraction treatment in osteoarthritis, it is important to acknowledge its limitations. Firstly, it is challenging to delve into the intricate details of joint distraction treatment strategies, including specifics on mechanical parameters, treatment duration, and their impact on therapeutic outcomes for osteoarthritis. Secondly, there may be a potential bias in the selection of publications due to the limitations of the chosen databases and language constraints. For instance, publications from renowned sources like Cochrane, Embase, and non-English language journals may not have been included. Lastly, there is a possibility that the most recent high-quality papers may not have garnered sufficient citations yet, potentially creating a gap between the findings of the bibliometric analysis and real-world developments. Therefore, we recommend that researchers remain vigilant for the latest publications, particularly those in non-English languages, to ensure a comprehensive and up-to-date understanding of the field.

## 5 Conclusion

In conclusion, we conduct this study to demonstrate status and global trends in joint distraction treatment in osteoarthritis research from 2003 to 2023. This study systematically showed the global trends and helped researchers identify the influential authors, institutions, and journals in this field. United States contributes the most publications, highest H-index, and citations in this area. In addtion, the keyword and co-citation clustering analysis also enable researchers to handle research directions mainly in four directions as follows “joint distraction techniques,” “distraction arthroplasty,” “joint repair mechanisms”, and “combinational surgical treatments.” We can expect that researchers can gain an in-depth understanding of current studies from this bibliometric and visualized analysis, and such understanding will be helpful for further investigations into this promising research field.

## Data Availability

The raw data supporting the conclusion of this article will be made available by the authors, without undue reservation.
