# Supplementary material for: Harnessing joint distraction for the treatment of osteoarthritis: a bibliometric and visualized analysis
Source: Front Bioeng Biotechnol. 2023 Nov 9;11:1309688. doi: 10.3389/fbioe.2023.1309688 (PMC10666289; doi:10.3389/fbioe.2023.1309688)
Supplement: Supplementary file 1 [file Table1.DOCX]

Supporting Information for

**Harnessing joint distraction for the treatment of osteoarthritis: A bibliometric and visualized analysis**

Liqing Peng^1†^, Runmeng Li^2†^, Shengxi Xu^1^, Keyuan Ding^1^, Yan Wu^1^, Hao Li^2^*, Yong Wang^1^*

^1^Department of Orthopedics, First People’s Hospital of Shuangliu District, No. 120, Chengbeishang Street, Shuangliu District, Chengdu 610200, China.

^2^School of Medicine, Nankai University, No. 94, Weijin Road, Nankai District, Tianjin 300071, China

†: These authors have contributed equally to this work.

*: Corresponding authors:

Email: Yongwang20150111@163.com, [lihao_DO@163.com](mailto:lihao_DO@163.com)

## Data Acquisition and Search Strategies

The acquisition of publications related to research on joint distraction in osteoarthritis treatment was undertaken using the SCI-Expanded database within the Web of Science Core Collection (WoSCC) from Clarivate Analytics. The search parameters were set from August 1, 2003, to August 1, 2023, and the search formula was structured as follows: TS= (osteoarthritis OR degenerative arthritis) AND TS= (distraction OR distractor OR traction OR arthrodiastasis). Moreover, the publication criteria were delineated as follows: (1) The publications predominantly centered on the theme of joint distraction in osteoarthritis treatment; (2) Document types were restricted to Articles and Reviews. (3) Papers were required to be composed in English. The exclusion criteria were likewise specified as follows: (1) Themes were not aligned with joint distraction in osteoarthritis treatment; (2) Publications classified as meeting abstracts, proceedings papers, corrections, book chapters, letters, news, and the like were excluded (see Figure 1). A meticulous evaluation of these publications was conducted by two reviewers (LQP and RML), with any publications deemed irrelevant to the research topic of joint distraction in osteoarthritis treatment being manually filtered out. Additionally, consultations with experienced corresponding authors were undertaken to adjudicate on whether to incorporate any potentially relevant but initially excluded publications into the present study.

**Table S1. Relevant articles category.**

|  | Number |
| --- | --- |
| Strongly relevant | 53 |
| Relatively relevant |  |
| I. Novel joint distraction techniques | 110 |
| II. Distraction arthroplasty | 124 |
| III. Joint repair mechanisms | 86 |
| IV. Joint diseases adjuvant applications | 96 |

**Table S2. Detailed title sheet for various groups**.

| **Strongly relevant** |
| --- |
| A pilot feasibility study for immediate relief of referred knee pain by hip traction in hip osteoarthritis |
| A prospective case-control study to compare the sensitivity and specificity of the grind and traction-shift (subluxation-relocation) clinical tests in osteoarthritis of the thumb carpometacarpal joint |
| A randomized controlled trial on the efficacy of intermittent and continuous traction for patients with knee osteoarthritis |
| Ankle Distraction Arthroplasty Combined with Joint Resurfacing for Management of an Osteochondral Defect of the Talus and Concomitant Osteoarthritis: A Case Report |
| Artificial intelligence in osteoarthritis: repair by knee joint distraction shows association of pain, radiographic and immunological outcomes |
| Basal Joint Osteoarthritis of the Thumb: Ligament Reconstruction and Tendon Interposition Versus Hematoma Distraction Arthroplasty |
| Biomechanical effectiveness of a distraction-rotation knee brace in medial knee osteoarthritis: Preliminary results |
| Can we decrease the duration of basal thumb joint distraction for early osteoarthritis from 8 to 6 weeks? Study protocol for a non-inferiority randomized controlled trial |
| Clinical and cost-effectiveness of Knee Arthroplasty versus Joint Distraction for Osteoarthritis (KARDS): protocol for a multicentre, phase III, randomised control trial |
| Clinical effectiveness and safety of a distraction-rotation knee brace for medial knee osteoarthritis |
| Clinical outcomes of knee joint distraction combined with marrow stimulation procedures for patients with advanced knee osteoarthritis |
| Comparisons between ankle distraction arthroplasty and supramalleolar osteotomy for treatment of post-traumatic varus ankle osteoarthritis |
| Comprehensive application of high tibial osteotomy, chronic distraction tissue regeneration, and computer-assisted external fixation in the treatment of severe knee osteoarthritis: A case report |
| Debridement and hinged motion distraction is superior to debridement alone in patients with ankle osteoarthritis: a prospective randomized controlled trial |
| Distraction arthroplasty in the management of osteoarthritis of the ankle: A systematic review |
| Distraction index as a risk factor for osteoarthritis associated with hip dysplasia in four large dog breeds |
| Distraction to treat knee osteoarthritis |
| Effectiveness of distal tibial osteotomy with distraction arthroplasty in varus ankle osteoarthritis |
| Evidence of Cartilage Repair by Joint Distraction in a Canine Model of Osteoarthritis |
| External Rotation Strengthening With Manual Distraction for Individuals With Glenohumeral Osteoarthritis |
| Functional analysis of distraction arthroplasty in the treatment of ankle osteoarthritis |
| Gene Expression Signatures of Synovial Fluid Multipotent Stromal Cells in Advanced Knee Osteoarthritis and Following Knee Joint Distraction |
| Hematoma and distraction arthroplasty for thumb basal joint osteoarthritis: Minimum 6.5-year follow-up evaluation |
| Initial tissue repair predicts long-term clinical success of knee joint distraction as treatment for knee osteoarthritis |
| Intermediate-Term Follow-up After Ankle Distraction for Treatment of End-Stage Osteoarthritis |
| Is Knee Joint Distraction a Viable Treatment Option for Knee OA? - A Literature Review and Meta-Analysis |
| Joint distraction attenuates osteoarthritis by reducing secondary inflammation, cartilage degeneration and subchondral bone aberrant change |
| Joint distraction for knee osteoarthritis: protocol for a systematic review and meta-analysis |
| Joint distraction for osteoarthritis: clinical evidence and molecular mechanisms |
| Joint distraction for thumb carpometacarpal osteoarthritis: a feasibility study with 1-year follow-up |
| Joint distraction using a purpose-built device for knee osteoarthritis |
| Knee Joint Distraction as Treatment for Osteoarthritis Results in Clinical and Structural Benefit: A Systematic Review and Meta-Analysis of the Limited Number of Studies and Patients Available |
| Knee Joint Distraction Compared to Total Knee Arthroplasty for Treatment of End Stage Osteoarthritis: Simulating Long-Term Outcomes and Cost-Effectiveness |
| Knee joint distraction in regular care for treatment of knee osteoarthritis: A comparison with clinical trial data |
| Mechanical traction from different knee joint angles in patients with knee osteoarthritis: A randomized controlled trial |
| Motion Versus Fixed Distraction of the Joint in the Treatment of Ankle Osteoarthritis: A Prospective Randomized Controlled Trial |
| Osteoarthritis of the Fourth and Fifth Tarsometatarsal Joint After Calcaneo-Cuboid Distraction Arthrodesis in Flatfoot Correction |
| Patient Characteristics as Predictors of Clinical Outcome of Distraction in Treatment of Severe Ankle Osteoarthritis |
| Patients with advanced lateral osteoarthritis can return to sports and work after distraction arthroplasty plus lateral meniscal allograft transplantation combined with cartilage repair |
| Prolonged clinical benefit from joint distraction in the treatment of ankle osteoarthritis |
| Simultaneous arthrodiastasis and deformity correction for a patient with ankle osteoarthritis and lower limb deformity: A case report |
| Six weeks of continuous joint distraction appears sufficient for clinical benefit and cartilaginous tissue repair in the treatment of knee osteoarthritis |
| Subchondral bone changes after joint distraction treatment for end stage knee osteoarthritis |
| Subchondral bone remodeling is related to clinical improvement after joint distraction in the treatment of ankle osteoarthritis |
| Supramalleolar Osteotomy With Distraction Arthroplasty in Treatment of Varus Ankle Osteoarthritis With Large Talar Tilt Angle: A Case Report and Literature Review |
| Supramalleolar osteotomy with medial distraction arthroplasty for ankle osteoarthritis with talar tilt |
| Sustained clinical and structural benefit after joint distraction in the treatment of severe knee osteoarthritis |
| Synovial fluid hyaluronan mediates MSC attachment to cartilage, a potential novel mechanism contributing to cartilage repair in osteoarthritis using knee joint distraction |
| Technical feasibility of personalized articulating knee joint distraction for treatment of tibiofemoral osteoarthritis |
| The molecular profile of synovial fluid changes upon joint distraction and is associated with clinical response in knee osteoarthritis |
| The Role of Distraction Arthroplasty in Managing Ankle Osteoarthritis |
| Tissue structure modification in knee osteoarthritis by use of joint distraction: an open 1-year pilot study |
| Unloading joints to treat osteoarthritis, including joint distraction |
|  |
| **I: novel joint distraction techniques** |
| 3.0 T MR imaging of the ankle: Axial traction for morphological cartilage evaluation, quantitative T2 mapping and cartilage diffusion imaging-A preliminary study |
| A new articulated distraction arthroplasty device for treatment of the osteoarthritic knee joint: A preliminary report |
| A new distraction arthroplasty device using magnetic force; a cadaveric study |
| A new distractor with angle-scale for proximal tibia medial opening wedge osteotomy |
| A novel 3D-printed patient-specific instrument based on "H-point" for medial opening wedge high tibial osteotomy: a cadaver study |
| Accuracy of 3D dual echo steady state (DESS) MR arthrography to quantify acetabular cartilage thickness |
| An alternative treatment method to restore limb-length discrepancy in osteoarthritis with high congenital hip dislocation |
| Ankle joint distraction arthroplasty for severe ankle arthritis |
| Anterograde removal of broken femoral nails without opening the nonunion site: a new technique |
| Assessment of the medium- to long-term radiographically confirmed outcome for juvenile dogs with hip dysplasia treated with double pelvic osteotomy |
| Canine hip dysplasia treated by juvenile pubic symphysiodesis Part I: Two year results of computed tomography and distraction index |
| Canine hip dysplasia treated by juvenile public symphysiodesis Part II: Two year clinical results |
| Cartilage Quality (dGEMRIC Index) Following Knee Joint Distraction or High Tibial Osteotomy |
| Cartilage regeneration for treatment of osteoarthritis: a paradigm for nonsurgical intervention |
| Clinical and radiological midterm outcome after treatment of pilonoidal fracture dislocations of the proximal interphalangeal joint with a parabolic dynamic external fixator |
| Clinical outcomes after arthroscopic acetabular labral repair using knot-tying or knotless suture technique |
| Comparison of distraction arthroplasty alone versus combined with arthroscopic microfracture in the treatment of posttraumatic ankle arthritis |
| Comparison of Short-Term Effects of Mobilization with Movement and Kinesiotaping on Pain, Function, and Balance in Patellofemoral Pain |
| Comparison of two radiographic techniques for evaluation of hip joint laxity in 10 breeds of dogs |
| Conventional and Arthrographic Magnetic Resonance Techniques for Hip Evaluation: What the Radiologist Should Know |
| Correction of tibial deformity in Paget's disease using the Taylor spatial frame |
| Correlation of Articular Cartilage Thickness Measurements Made with Magnetic Resonance Imaging, Magnetic Resonance Arthrography, and Computed Tomographic Arthrography with Gross Articular Cartilage Thickness in the Equine Metacarpophalangeal Joint |
| Cost Analysis and National Trends in the Treatment of Thumb Basal Arthritis Comparing Ligament Reconstruction/Tendon Interposition and Trapeziectomy/Hematoma Distraction Arthroplasty |
| Current Advancements in Ankle Arthrodiastasis |
| Development and Assessment of a Microcomputed Tomography Compatible Five Degrees-of-Freedom Knee Joint Motion Simulator |
| Development of a clinical prediction rule to identify patients with knee pain and clinical evidence of knee osteoarthritis who demonstrate a favorable short-term response to hip mobilization |
| Distal Tibial Varus |
| Distraction Arthroplasty With Arthroscopic Microfracture in a Patient With Rheumatoid Arthritis of the Ankle Joint |
| Distraction index measurement on the dog's hip joint using a dedicated software |
| Double Tightrope for Basilar Thumb Arthritis |
| Dr. Buzby's ToeGrips® Application Results in Minimal Changes in Kinetic Gait Parameters in Normal Dogs |
| Dynamic external traction system for management of distal displaced fractures of the head of the proximal phalanx |
| Dynamic interspinous process technology |
| Effect of high tibial flexion osteotomy on cartilage pressure and joint kinematics: a biomechanical study in human cadaveric knees - Winner of the AGA-DonJoy Award 2004. |
| Eliminating senescent chondrogenic progenitor cells enhances chondrogenesis under intermittent hydrostatic pressure for the treatment of OA |
| Enhanced tibial osteotomy healing with use of bone grafts supplemented with platelet gel or platelet gel and bone marrow stromal cells. |
| Evaluation of gait kinetics in puppies with coxofemoral joint laxity |
| Evaluation of multiple radiographic predictors of cartilage lesions in the hip joints of eight-month-old dogs |
| Evidences of the use of cattle as draught animals in Chalcolithic of El Portalon (Sierra de Atapuerca, Burgos) |
| Experimental Study on the Prevention of Posttraumatic Osteoarthritis in the Rabbit Knee Using a Hinged External Fixator in Combination with Exercises |
| Extension osteotomy of the metacarpal I and ligamentoplasty of the trapeziometacarpal joint for the treatment of early-stage osteoarthritis and instability of the trapeziometacarpal joint |
| External fixation-assisted reduction for the treatment of neglected hip dislocations with limb length discrepancy: a retrospective study of 13 cases |
| Fibular Lengthening Using Distraction Osteogenesis for the Treatment of Fibular Malunion: A Case Report |
| Finite Element Analysis and Physiologic Testing of a Novel, Inset Glenoid Fixation Technique |
| Freiberg's Infraction of the Second Metatarsal Treated with Autologous Osteochondral Transplantation and External Fixation |
| Functional outcome with special attention to the DASH questionnaire following callus distraction and phalangization of the thumb after traumatic amputation in the middle one-third |
| Greater frequency of distraction accelerates bone formation in open-wedge proximal tibial osteotomy with hemicallotasis |
| Hemicallotasis for Correction of Varus Deformity of the Proximal Tibia Using a Unilateral External Fixator |
| High tibial osteotomy with use of the Taylor Spatial Frame external fixator for osteoarthritis of the knee |
| Ilizarov Gradual Distraction Correction for Distal Tibial Severe Varus Deformity Resulting from Epiphyseal Fracture: Case Report and Literature Review |
| Instructive cartilage regeneration modalities with advanced therapeutic implantations under abnormal conditions |
| Interposition Ankle Arthroplasty Using Achilles Tendon Allograft (The AAA Procedure): A Case Report |
| Intra-articular nerve growth factor regulates development, but not maintenance, of injury-induced facet joint pain & spinal neuronal hypersensitivity |
| Intraoperative soft tissue balance using novel medial preserving gap technique in posterior-stabilized total knee arthroplasty: comparison to measured resection technique |
| Is a hip distractor useful in the arthroscopic treatment of femoroacetabular impingement? |
| Joint distraction and movement for repair of articular cartilage in a rabbit model with subsequent weight-bearing |
| Joint Preservation Procedures for Ankle Arthritis |
| Knee arthritis in congenital short femur after Wagner lengthening |
| Large Versus Small Opening Wedge High Tibial Osteotomies Performed With a Protective Wire Over the Lateral Hinge: Incidence of Lateral Hinge Fracture and Early Clinical Outcomes |
| Management of Osteoarthrosis of the Thumb Joints |
| Minimally Invasive Osteosynthesis Technique for Articular Fractures |
| Nail-medullary canal ratio affects mechanical axis deviation during femoral lengthening with an intramedullary distractor |
| Osteotome axial twist (OAT) technique: A simple method for exposing arthritic foot joints in preparation for arthrodesis |
| Other surgical techniques for osteoarthritis |
| Outcome After Modified Grice-Green Procedure (SAMBB) for Arthritic Acquired Adult Flatfoot |
| Outcome After Operative Fusion of the Tarsal Joints: A Systematic Review |
| Preoperative factors associated with extension gap in cruciate-retaining total knee arthroplasty: A retrospective study on continuous determination of distraction force |
| Primary osteoarthritis of the elbow: Current treatment options |
| Prognostic value of chondral defects on the outcome after arthroscopic treatment of acetabular labral tears |
| Progressive Junctional Kyphosis at the Caudal End of Lumbar Instrumented Fusion: Etiology, Predictors, and Treatment |
| Protocol development for synchrotron contrast-enhanced CT of human hip cartilage. |
| Provisional heritability estimates of four distraction index traits in a breeding population of German Shepherd dogs |
| Proximal interphalangeal joint fractures treated with a dynamic external fixator: A multicenter and retrospective study of 88 cases. |
| Reference ranges for atlantodental interval in adults and its variation with age and gender in a large series of subjects on multidetector computed tomography. |
| Reinforcement strategy for medial open-wedge high tibial osteotomy: a finite element evaluation of the additional opposite screw technique and bone grafts. |
| Relationships among measurements obtained by use of computed tomography and radiography and scores of cartilage microdamage in hip joints with moderate to severe joint laxity of adult dogs |
| Reminiscence About Physical Activity A Pilot Study to Reduce Pain in Older Adults |
| Return to Sport and Work after Randomization for Knee Distraction versus High Tibial Osteotomy: Is There a Difference? |
| Review of thumb carpometacarpal arthritis classification, treatment and outcomes |
| Rheumatologic illnesses - Treatment strategies for older adults |
| Risk factors for failure of reduction of anterior glenohumeral dislocation without sedation. |
| Rotational Acetabular Osteotomy through an Ollier Lateral U Approach for Early-stage Osteoarthritis Secondary to Acetabular Dysplasia |
| Sagittal Reconstruction of the Atlantoaxial Lateral Mass Complex with an Intra-Articular Cage Fusion Technique for Degenerative Atlantoaxial Instability. |
| Severe slipped capital femoral epiphysis: A French multicenter study of 186 cases performed by the SoFOP |
| Sport and physical activity in patients after derotational corticotomies with the Ilizarov method |
| Squatting, lunging and kneeling provided similar kinematic profiles in healthy knees - A systematic review and meta-analysis of the literature on deep knee flexion kinematics. |
| Subtalar distraction arthroplasty with bone marrow aspirate concentrate (BMAC), preliminary results of a new joint preservation technique |
| Suture Button Compared With K-Wire Fixation for Maintenance of Posttrapeziectomy Space Height in a Cadaver Model of Lateral Pinch |
| Temporary Segmental Distraction in a Dog with Degenerative Lumbosacral Stenosis |
| Temporomandibular Joint Reconstruction in the Growing Child |
| The "Pillow" Technique for Thumb Carpometacarpal Joint Arthritis: Cohort Study With 10-to 15-Year Follow-Up |
| The effect of a single high velocity low amplitude hip mobilization on strength in subjects with knee injuries |
| The effectiveness of peripheral compartment first access and periportal capsulotomy technique for arthroscopic management of femoroacetabular impingement: A prospective case series |
| The Hip Suction Seal, Part II: The Effect of Rim Trimming, Chondrolabral Junction Separation, and Labral Repair/Refixation on Hip Distractive Stability. |
| The Influence of Gardening Activities on Self-reported Health Problems, Allergies, and Body Mass Index |
| The optimal injection technique for the osteoarthritic ankle: A randomized, cross-over trial |
| The Use of Virtual Planning and Navigation in the Treatment of Temporomandibular Joint Ankylosis |
| Three-Dimensional Assessment of the Influence of Juvenile Pubic Symphysiodesis on the Pelvic Geometry of Dogs |
| Three-Dimensional Kinematic Evaluation of Lateral Suture Stabilization in an In Vitro Canine Cranial Cruciate Deficient Stifle Model |
| Tibio-talo-calcaneal fusion after limb salvage procedures-A retrospective study. |
| TMJ energy densities in healthy men and women |
| Towards a Diagnostic Tool for Diagnosing Joint Pathologies: Supervised Learning of Acoustic Emission Signals |
| Trapeziectomy |
| Trapeziectomy for trapeziometacarpal joint osteoarthritis: is ligament reconstruction and temporary stabilisation of the pseudarthrosis with a Kirschner wire important? |
| Ultrasonic and radiographic study of laxity in hip joints of young dogs |
| Update on Thumb Basal Joint Arthritis Surgery |
| Using a finite element model of the thumb to study Trapeziometacarpal joint contact during lateral pinch |
| Value of Examination Under Fluoroscopy for the Assessment of Sacroiliac Joint Dysfunction |
| What Are the Predictors of Self-Reported Change in Physical Activity in Older Adults with Knee or Hip Osteoarthritis? |
| What's New in Limb Lengthening and Deformity Correction |
|  |
| **II: Distraction arthroplasty** |
| A convex lateral tibial plateau for knee replacement |
| A pilot randomized controlled trial of flexion-distraction dosage for chiropractic treatment of lumbar spinal stenosis |
| A Systematic Review of Postoperative Hand Therapy Management of Basal Joint Arthritis |
| Acetabular fracture: Long-term follow-up and factors associated with secondary implantation of total hip arthroplasty |
| All-Arthroscopic Autologous Matrix-Induced Chondrogenesis-Aided Repair of a Patellar Cartilage Defect Using Dry Arthroscopy and a Retraction System |
| Ankle Arthrodiastasis and Interpositional Ankle Exostectomy |
| Ankle Arthrodiastasis with Circular External Fixation for the Treatment of Posttraumatic Ankle Arthritis |
| Ankle arthroplasty alternatives with allograft and external fixation: Preliminary clinical outcome |
| Ankle Arthroscopy: An Update |
| Ankle distraction arthroplasty for the treatment of severe ankle arthritis Case report, technical note, and literature review |
| Ankle Distraction Arthroplasty: Indications, Technique, and Outcomes |
| Ankle Joint Distraction Arthroplasty: Why and How? |
| Ankle joint distraction is a promising alternative treatment for patients with severe haemophilic ankle arthropathy |
| Anterolateral muscle sparing approach total hip arthroplasty: an anatomic and clinical study. |
| Arthroscopic Ankle Arthrodesis: Factors Influencing Union in 39 Consecutive Patients |
| Arthroscopic partial limbectomy in hip joints with acetabular hypoplasia |
| Arthroscopic treatment of femoroacetabular impingement: Technical review |
| Arthroscopic treatment of femoroacetabular pincer impingement |
| Articulated Joint Distraction in a Cadaveric Model of the Canine Elbow |
| Canine Hip Dysplasia Diagnostic Imaging |
| Canine Hip Dysplasia Screening Within the United States: Pennsylvania Hip Improvement Program and Orthopedic Foundation for Animals Hip/Elbow Database |
| Canine hip dysplasia: phenotypic scoring and the role of estimated breeding value analysis. |
| Changes in Cartilage Thickness and Denuded Bone Area after Knee Joint Distraction and High Tibial Osteotomy-Post-Hoc Analyses of Two Randomized Controlled Trials |
| Chronology of Hip Dysplasia Development in a Cohort of 48 Labrador Retrievers Followed for Life |
| Comparison of high, medium and low mobilization forces for increasing range of motion in patients with hip osteoarthritis: A randomized controlled trial |
| Comparison of high, medium and low mobilization forces for reducing pain and improving physical function in patients with hip osteoarthritis: Secondary analysis of a randomized controlled trial. |
| Complex Fractures of the Acetabulum: Should the Enlarged Iliofemoral Approach Be Abandoned? Results at 20 Years' Follow-up |
| Correlation Between Intraoperative Anterior Stability and Flexion Gap in Total Knee Arthroplasty |
| Correlation of Obesity With Patient-Reported Outcomes and Complications After Hip Arthroscopy |
| Correlation of urinary nitric oxide concentrations with the development of hip dysplasia in labrador retrievers |
| Decreased Synovial Inflammation in Atraumatic Hip Microinstability Compared With Femoroacetabular Impingement |
| Distally based capsule-periosteum interpositional arthroplasty for hallux rigidus - Indications, operative technique, and short-term follow-up |
| Distraction arthroplasty of the trapeziometacarpal joint |
| Dysplasia of the contralateral hip in patients with unilateral late-detected congenital dislocation of the hip 50 YEARS' FOLLOW-UP OF 48 PATIENTS. |
| Early morbidity and mortality after one-stage bilateral THA: Anterior versus posterior approach. |
| Effect of stabilization splint therapy on glenoid fossa remodeling in temporomandibular joint osteoarthritis |
| Etiology of the anterior ankle impingement syndrome: A descriptive anatomical study |
| Evaluation of a self-retaining distractor for hip joint arthroscopy in toy breed dogs |
| Evaluation of Quantitative Trait Loci for Hip Dysplasia in Labrador Retrievers |
| Extended Fixation for Paprosky Type III Acetabular Defects in Revision Total Hip Arthroplasty with a Minimum Follow-Up of 2 Years |
| Five-year followup of ankle joint distraction for post-traumatic chondrolysis in an adolescent: A case report |
| Five-Year Follow-up of Knee Joint Distraction: Clinical Benefit and Cartilaginous Tissue Repair in an Open Uncontrolled Prospective Study |
| Fracture-separation of the distal humeral epiphysis - Long-term follow-up of five cases |
| Glenohumeral mismatch affects micromotion of cemented glenoid components in total shoulder arthroplasty |
| Hallux Rigidus: What Lies Beyond Fusion, Resectional Arthroplasty, and Implants |
| Hinged External Fixation Distraction for Treatment of First Metatarsophalangeal Joint Arthritis |
| Hip Dysplasia Clinical Signs and Physical Examination Findings |
| Hyperextension-distraction fractures in ankylosing and spondylotic spines: injury profile and treatment results |
| Immediate effects of manual traction on radiographically determined joint space width in the hip joint |
| Ipsilateral total hip arthroplasty in patient with an above-knee amputee for femoral neck fracture: a case report |
| Is There Any Value to Arthroscopic Debridement of Ankle Osteoarthritis and Impingement? |
| Knee Joint Distraction Compared with High Tibial Osteotomy and Total Knee Arthroplasty: Two-Year Clinical, Radiographic, and Biochemical Marker Outcomes of Two Randomized Controlled Trials |
| Knee joint distraction compared with high tibial osteotomy: a randomized controlled trial |
| Knee joint distraction compared with total knee arthroplasty: A randomized controlled trial |
| Knee joint distraction results in MRI cartilage thickness increase up to 10 years after treatment |
| Large Osteochondral Defects of the Distal Tibia Plafond After Septic Arthritis of the Ankle Joint Treated by Arthrodiastasis and Iliac Bone Graft: A Case Report |
| Late Reduction in Congenital Dislocation of the Hip and the Need for Secondary Surgery: Radiologic Predictors and Confounding Variables. |
| Leg Length Discrepancy - Treatment Indications and Strategies |
| Long-Term Follow-Up of Basal Joint Resection Arthroplasty of the Thumb With Transfer of the Abductor Pollicis Brevis Origin to the Flexor Carpi Radialis Tendon |
| Long-term outcome of closed reduction in late-detected hip dislocation: 60 patients aged six to 36 months at diagnosis followed to a mean age of 58 years |
| Management and Outcomes of Hip Fractures in Lower Limb Amputees: A Case Series |
| Manual therapy intervention for a patient with a total hip arthroplasty revision |
| Medial compartment osteoarthritis of the knee: a review of surgical options |
| New Technologies in Knee Arthroplasty: Current Concepts |
| Opioid use in knee or hip osteoarthritis: a region-wide population-based cohort study |
| Outcomes of hip arthroscopy. A prospective analysis and comparison between patients under 25 and over 25 years of age |
| Palpation and Dorsal Acetabular Rim Radiographic Projection for Early Detection of Canine Hip Dysplasia: A Prospective Study |
| Percutaneous fixation of acetabular fractures |
| Plantar calcaneal spurs in older people: longitudinal traction or vertical compression? |
| Postoperative Knee Flexion Angle Is Affected by Lateral Laxity in Cruciate-Retaining Total Knee Arthroplasty |
| Posttraumatic ankle arthritis: An update on conservative and surgical management |
| Prediction of cartilaginous tissue repair after knee joint distraction |
| Preliminary Analysis of Adjacent Segment Degeneration in Patients Treated with Posterior Cervical Cages: 2-Year Follow-Up. |
| Primary or Secondary Subtalar Arthrodesis and Revision of Calcaneal Nonunion with Minimally Invasive Rigid Internal Nail Fixation for Treatment of Displaced Intra-Articular Calcaneal Fractures |
| Principal component analysis of canine hip dysplasia phenotypes and their statistical power for genome-wide association mapping |
| Puppy line, metaphyseal sclerosis, and caudolateral curvilinear and circumferential femoral head osteophytes in early detection of canine hip dysplasia. |
| Realignment Surgery for Malunited Ankle Fracture |
| Relatively Loose Flexion Gap Improves Patient-Reported Clinical Scores in Cruciate-Retaining Total Knee Arthroplasty. |
| Revision of the Failed Thumb Carpometacarpal Arthroplasty |
| Revision of trapeziectomy failures in thumb base osteoarthritis: retrospective analysis of ten cases and review of the literature |
| Similar Healthcare Utilization and 1-Year Patient-Reported Outcomes Between Cemented and Cementless Primary Total Knee Arthroplasty: A Propensity Score-Matched Analysis |
| Simultaneous Versus Staged Bilateral Hip Arthroscopy in the Treatment of Femoroacetabular Impingement |
| Standard versus physiologic bone preparation in total knee arthroplasty and the effect on joint space opening. |
| Subscapularis function after total shoulder arthroplasty: electromyography, ultrasound, and clinical correlation. |
| Subtalar Joint Arthrodesis for Elective and Posttraumatic Foot and Ankle Deformities |
| Supramalleolar Osteotomy and Ankle Arthrodiastasis for Juvenile Posttraumatic Ankle Arthritis |
| Surgery for trapeziometacarpal osteoarthritis |
| Surgical interventions for symptomatic knee osteoarthritis: a network meta-analysis of randomized control trials |
| Surgical interventions for symptomatic mild to moderate knee osteoarthritis |
| Surgical procedures in patients with haemophilic arthropathy of the ankle |
| Surgical Technique and Case Series of Total Hip Arthroplasty with the Hueter Anterior Approach for Crowe Type-IV Dysplasia |
| Surgical Treatment of Advanced Carpometacarpal Joint Arthritis Trapeziectomy with Hematoma Arthroplasty |
| Talar OsteoPeriostic grafting from the Iliac Crest (TOPIC) for lateral osteochondral lesions of the talus: operative technique |
| The Concept of Ankle Joint Preserving Surgery: Why Does Supramalleolar Osteotomy Work and How to Decide When to Do an Osteotomy or Joint Replacement |
| The contribution of intraoperative medial compartment stability to post-operative knee flexion angle in unicompartmental knee arthroplasty. |
| The Direct Anterior Approach for Total Hip Arthroplasty for Severe Dysplasia (Crowe III and IV) Provides Satisfactory Medium to Long-Term Outcomes |
| The effect of glenohumeral radial mismatch on different augmented total shoulder arthroplasty glenoid designs: a finite element analysis |
| The effect of tibio-femoral over-distraction in primary knee arthroplasty |
| The flexion-extension gap is predictive of patient-reported outcome measures after cruciate-retaining total knee arthroplasty |
| The hip antero-superior labral tear with avulsion of rectus femoris (HALTAR) lesion: does the SLAP equivalent in the hip exist? |
| The Learning Curve for Hip Arthroscopic Surgery: A Prospective Evaluation With 2-Year Outcomes in Patients With Femoroacetabular Impingement. |
| The posterior cervical transdural approach for retro-odontoid mass pseudotumor resection: report of three cases and discussion of the current literature. |
| The Role of Anterior Ankle Arthroscopy in the Management of Ankle Arthritis Literature Review, Patient Evaluation, Goals of Treatment and Technique |
| The Role of Arthroscopy in Trapeziometacarpal Arthritis |
| The tibia first technique with tensor measurement is useful to predict the soft tissue tension after implantation in unicompartmental knee arthroplasty. |
| Three-Dimensional Distribution of Acetabular Cartilage Thickness in Patients with Hip Dysplasia: A Fully Automated Computational Analysis of MR Imaging |
| Thumb Basal Joint Arthroplasty Using Abductor Pollicis Longus Tendon: An Average 5.5-Year Follow-Up. |
| Tibial fractures in children: A retrospective 27-year follow-up study |
| Tibial lengthening for unilateral Crowe type-IV developmental dysplasia of the hip. |
| Total Hip Arthroplasty for Osteoarthritis in Patients Aged 80 Years or Older: Influence of Co-morbidities on Final Outcome |
| Total trapeziectomy |
| Trapezium Resection With Suspension and Interposition Arthroplasty Using Acellular Dermal Allograft for Thumb Carpometacarpal Arthritis. |
| Treatment of advanced carpometacarpal joint disease: Trapeziectomy and hematoma arthroplasty |
| Treatment of Advanced Kienbock Disease With a Vascularized Radial Bone Flap Wrapped in the Pronator Quadratus |
| Treatment of proximal interphalangeal joint fractures by the pins and rubbers traction system: a follow-up |
| Treatment of tibial plateau fractures: A comparison of two different operation strategies with medium-term follow up |
| Trends in the phenotypic hip status of selected breeds of dog as measured by the New Zealand Veterinary Association Hip Dysplasia scheme (1990-2008). |
| Tuberculosis of hip in children: A retrospective analysis. |
| Two Different Total Hip Arthroplasties for Hartofilakidis Type C1 Developmental Dysplasia of Hip in Adults. |
| Uncemented Total Hip Arthroplasty Through the Direct Anterior Approach: Analysis of a Consecutive Series of 275 Hips With a Minimum Follow-Up of 10 Years |
| Unstable slipped capital femoral epiphysis: reduction by gradual distraction with external fixator. A case report |
| Update to femoroacetabular impingement arthroscopic management |
| Value of arthroscopy in the treatment of upper ankle arthritis |
| Visualization of the distal tibial plafond articular surface using four established approaches and the efficacy of instrumented distraction: a cadaveric study |
|  |
| **III: Joint repair mechanisms** |
| A Novel Arthroscopic Technique for Intraoperative Mobilization of Synovial Mesenchymal Stem Cells. |
| A novel hypothesis: The application of platelet-rich plasma can promote the clinical healing of white-white meniscal tears |
| An Alternative Patellar Fracture Fixation: A Biomechanical Study |
| An in vitro analysis of ligament reconstruction or extension osteotomy on trapeziometacarpal joint stability and contact area |
| Articular Cartilage Regeneration and Tissue Engineering Models: A Systematic Review |
| Assessment of Sensory Thresholds in Dogs using Mechanical and Hot Thermal Quantitative Sensory Testing |
| Associations between canine juvenile weight gain and coxofemoral joint laxity at 16 weeks of age |
| Associations between early radiographic and computed tomographic measures and canine hip joint osteoarthritis at maturity |
| Autologous Osteochondral Graft for Early Posttraumatic Arthritis of Tibiotalar Joints After Comminuted Pilon Fractures in Young Patients |
| Bilateral anterior elevation prosthesis boosts chondrocytes proliferation in mice mandibular condyle |
| Biomechanical evaluation contribution of the acetabular labrum to hip stability |
| Bipolar Fresh Osteochondral Allograft Transplantation of the Tibiotalar Joint |
| Bisphosphonate-coated external fixation pins appear similar to hydroxyapatite-coated pins in the tibial metaphysis and to uncoated pins in the shaft: A randomized trial. |
| Bone marrow edema in sports: General concepts |
| Carpometacarpal Joint Pathology in the Thumb and Hand Evaluation and Management of Difficult Conditions |
| Cartilage Repair Activity during Joint-Preserving Treatment May Be Accompanied by Osteophyte Formation |
| Cartilage thickness in the hip joint measured by MRI and stereology - a methodological study |
| Chronic Pain Characteristics and Gait in Older Adults: The MOBILIZE Boston Study II |
| Comparison between 2D radiographic weight-bearing joint space width and 3D MRI non-weight-bearing cartilage thickness measures in the knee using non-weight-bearing 2D and 3D CT as an intermediary |
| Comparison between in vitro and in vivo cartilage overloading studies based on a systematic literature review |
| Comparison of Cannulated Screws Versus Compression Staples for Subtalar Arthrodesis Fixation |
| Condylar repair and regeneration in adolescents/young adults with early-stage degenerative temporomandibular joint disease: A randomized controlled study |
| Craniovertebral instability due to degenerative osteoarthritis of the atlantoaxial joints: analysis of the management of 108 cases Clinical article |
| Current Concepts in the Management of Ankle Osteoarthritis: A Systematic Review |
| Current status and future prospects for disease modification in osteoarthritis |
| Differences in joint morphology between the knee and ankle affect the repair of osteochondral defects in a rabbit model. |
| Dysregulation of the Wnt Signaling Pathway and Synovial Stem Cell Dysfunction in Osteoarthritis Development |
| Effect of tibial coronal inclination on hindfoot kinematics: A biomechanical simulation study. |
| Effects of Low-Intensity Pulsed Ultrasound on Radiographic Healing of Tibial Plateau Leveling Osteotomies in Dogs: A Prospective, Randomized, Double-Blinded Study |
| Effects of osteoarthritis on radiographic measures of laxity and congruence in hip joints of Labrador Retrievers |
| Effects of ultrasound on estradiol level, bone mineral density, bone biomechanics and matrix metalloproteinase-13 expression in ovariectomized rabbits |
| End-Stage Ankle Arthritis Arthrodiastasis, Supramalleolar Osteotomy, or Arthrodesis? |
| Enhanced Extracellular Matrix Breakdown Characterizes the Early Distraction Phase of Canine Knee Joint Distraction |
| Etiopathogenesis of Canine Hip Dysplasia, Prevalence, and Genetics |
| Etiopathogenesis of osteoarthritis |
| Etiopathogenesis of Osteoarthritis |
| Evaluation of a fibrillin 2 gene haplotype associated with hip dysplasia and incipient osteoarthritis in dogs |
| Evidence-Based Indications for Distraction Ankle Arthroplasty |
| Evolution of Radiographic Parameters of Canine Passive Hip Laxity at 4, 6 and 12 months: A Study of 306 Dogs |
| Experimental Pain Is Alleviated by Manual Traction Itself Rather than Subjective Bias in the Knee: A Signal Detection Analysis |
| Fifth metacarpal instability and its effect on hamatometacarpal arthritis patterns-a cadaver study |
| First Carpometacarpal Joint Denervation: A Systematic Review |
| Functional articular cartilage repair: here, near, or is the best approach not yet clear? |
| Genetic improvement of hip-extended scores in 3 breeds of guide dogs using estimated breeding values: Notable progress but more improvement is needed |
| Genomic Prediction of Two Complex Orthopedic Traits Across Multiple Pure and Mixed Breed Dogs. |
| Hereditary bilateral genu recurvatum: Case report of a family |
| How do lateral hinge and distraction affect three-dimensional rotation in open wedge high tibial osteotomy? |
| Hypoalgesic and motor effects of kaltenborn mobilization on elderly patients with secondary thumb carpometacarpal osteoarthritis: a randomized controlled trial |
| In Vitro Evaluation of Small Molecule Delivery into Articular Cartilage: Effect of Synovial Clearance and Compressive Load |
| Joint distraction results in clinical and structural improvement of haemophilic ankle arthropathy: a series of three cases |
| Joint unloading implant modifies subchondral bone trabecular structure in medial knee osteoarthritis: 2-year outcomes of a pilot study using fractal signature analysis |
| K-wire transfixation or distraction following the Keller-Brandes arthroplasty in Hallux rigidus and Hallux valgus? |
| Less mechanical loading attenuates osteoarthritis by reducing cartilage degeneration, subchondral bone remodeling, secondary inflammation, and activation of NLRP3 inflammasome |
| Low-intensity pulsed ultrasound (LIPUS) and pulsed electromagnetic field (PEMF) treatments affect degeneration of cultured articular cartilage explants |
| Measuring physiotherapy performance in patients with osteoarthritis of the knee: A prospective study |
| Mechanism of reducing knee adduction moment by shortening of the knee lever arm via medio-lateral manipulation of foot center of pressure: A pilot study |
| Mechanotransduction and Stiffness-Sensing: Mechanisms and Opportunities to Control Multiple Molecular Aspects of Cell Phenotype as a Design Cornerstone of Cell-Instructive Biomaterials for Articular Cartilage Repair. |
| Nobiletin suppresses IL-21/IL-21 receptor-mediated inflammatory response in MH7A fibroblast-like synoviocytes (FLS): An implication in rheumatoid arthritis. |
| Of mice and men: converging on a common molecular understanding of osteoarthritis |
| Optimal Distraction Force for Evaluating Tibiofemoral Joint Gaps in Posterior Stabilized Total Knee Arthroplasty |
| Osteoarthritis and intervertebral disc degeneration: Quite different, quite similar |
| Osteoarthritis in year 2021: biochemical markers |
| Osteoarthritis year 2011 in review: clinical |
| Osteoarthritis Year in Review 2014: rehabilitation and outcomes |
| Osteophytes: The product of convergent evolution |
| Pain from intra-articular NGF or joint injury in the rat requires contributions from peptidergic joint afferents |
| Perspectives on chondrocyte mechanobiology and osteoarthritis |
| Physiotherapy for Patients with Hip and Knee Osteoarthritis in Germany: A Survey of Current Practice |
| Posterior cruciate ligament resection under minimum medial collateral ligament release changes tibial internal rotation, joint center gap, and varus ligament balance on joint distraction force at flexion in total knee arthroplasty |
| Prevalence of osteophytes associated with the acromion and acromioclavicular joint |
| Regulatory Mechanism of The Induction of Regulatory T Cells through Growth Factors Released by Human Mesenchymal Stem Cells |
| Relationship between individual forces of each quadriceps head during low-load knee extension and cartilage thickness and knee pain in women with knee osteoarthritis |
| Relationships of hip joint volume ratios with degrees of joint laxity and degenerative disease from youth to maturity in a canine population predisposed to hip joint osteoarthritis |
| Review Article: Osteophytes |
| Role of Alpha-2-Microglobulin in the Treatment of Osteoarthritic Knee Pain: a Brief Review of the Literature |
| Single nucleotide polymorphisms refine QTL intervals for hip joint laxity in dogs |
| Size-invariant descriptors for detecting regions of abnormal growth in cervical vertebrae |
| Subchondral plate porosity colocalizes with the point of mechanical load during ambulation in a rat knee model of post-traumatic osteoarthritis |
| Targeting mechanotransduction pathways in osteoarthritis: a focus on the pericellular matrix |
| The catabolic-to-anabolic shift seen in the canine osteoarthritic cartilage treated with knee joint distraction occurs after the distraction period |
| The effect of lateral cortex disruption and repair on the stability of the medial opening wedge high tibial osteotomy. |
| The effect of tibial lengthening on immature articular cartilage of the knee joint |
| The effect of translational and rotational traction on lower extremity joint loading |
| The Extracellular Matrix of Articular Cartilage Controls the Bioavailability of Pericellular Matrix-Bound Growth Factors to Drive Tissue Homeostasis and Repair |
| The role of low-intensity pulsed ultrasound on bone and soft tissue healing |
| The Roles of Mechanical Stresses in the Pathogenesis of Osteoarthritis: Implications for Treatment of Joint Injuries |
|  |
| **IV: Joint diseases adjuvant applications** |
| A comparison of intra-articular hyaluronan injection accuracy rates between three approaches based on radiographic severity of knee osteoarthritis |
| Abductor pollicis longus hemitendon looping around the first intermetacarpal ligament as interposition following trapeziectomy: A one-year follow-up study |
| An Evidence-Based Approach to Treating Thumb Carpometacarpal Joint Arthritis |
| Analysis and classification of gait patterns in osteoarthritic and asymptomatic knees using phase space reconstruction, intrinsic time-scale decomposition and neural networks |
| Ankle Arthritis Review of Diagnosis and Operative Management |
| Application of a New M-shaped High Tibial Osteotomy Method: A Technical Note |
| Assessment of the hip reduction angle for predicting osteoarthritis of the hip in the Labrador Retriever |
| Biomechanical Stability of High Tibial Opening Wedge Osteotomy: Internal Fixation Versus External Fixation |
| Bony abnormalities in classic bladder exstrophy: The urologist's perspective |
| Cartilage Thickness in the Hip Measured by MRI and Stereology Before and After Periacetabular Osteotomy |
| Chiropractic management of the kinetic chain for the treatment of hip osteoarthritis: an Australian case series |
| Clinical Experience Using a 3D-Printed Patient-Specific Instrument for Medial Opening Wedge High Tibial Osteotomy. |
| Clinical Outcomes for Neurogenic Claudication Using a Multimodal Program for Lumbar Spinal Stenosis: A Retrospective Study |
| Combination therapy with intra-articular injection of mesenchymal stem cells and articulated joint distraction for repair of a chronic osteochondral defect in the rabbit |
| Combined technique for the correction of lower-limb deformities resulting from metabolic bone disease |
| Comparative evaluation of posterior cruciate ligament in total knee arthroplasty: An in vivo study |
| Comparison of Intraoperative Soft Tissue Balance Between Cruciate-Retaining and Posterior-Stabilized Total Knee Arthroplasty Performed by a Newly Developed Medial Preserving Gap Technique |
| Comparison of Intraoperative Soft Tissue Balance Measurement Between Two Tensor Systems in Total Knee Arthroplasty |
| Comparison of the efficiency of an extra-articular absorber system and high tibial osteotomy for unloading the medial knee compartment: an in vitro study |
| Comparison of Three Methods to Quantify Laxity in the Canine Hip Joint |
| Comparison of Trapeziectomy and Trapeziectomy with Ligament Reconstruction and Tendon Interposition: A Systematic Literature Review |
| Correlation of distraction index with arthroscopic findings in juvenile dogs with hip dysplasia |
| Diagnostic Value of Computed Tomography and Risk Factors for Lateral Hinge Fracture in the Open Wedge High Tibial Osteotomy |
| Directing clinical care using lower extremity biomechanics in patients with ankle osteoarthritis and ankle arthroplasty |
| Discharging the medial knee compartment: comparison of pressure distribution and kinematic shifting after implantation of an extra-capsular absorber system (ATLAS) and open-wedge high tibial osteotomy-a biomechanical in vitro analysis. |
| Do Ahlback scores identify subgroups with different magnitudes of cartilage thickness loss in patients with moderate to severe radiographic osteoarthritis? One-year follow-up data from the Osteoarthritis Initiative |
| Does using the direct anterior approach with a standard table for total hip arthroplasty reduce leg length discrepancies? Comparative study of traction table versus standard table |
| Effect of Microfracture on Functional Outcomes and Subchondral Sclerosis Following Distraction Arthroplasty of the Ankle Joint |
| Effect of Perturbing a Simulated Motion on Knee and Anterior Cruciate Ligament Kinetics |
| Effective repair of a fresh osteochondral defect in the rabbit knee joint by articulated joint distraction following subchondral drilling |
| Efficacy of treating low back pain and dysfunction secondary to osteoarthritis: Chiropractic care compared with moist heat alone |
| Enhancement of Tendon-Bone Healing for Anterior Cruciate Ligament (ACL) Reconstruction Using Bone Marrow-Derived Mesenchymal Stem Cells Infected with BMP-2. |
| Evaluation of Articular Cartilage Injury Using Computed Tomography With Axial Traction in the Ankle Joint |
| Evaluation of joint laxity against distal traction force upon flexion in cruciate-retaining and posterior-stabilized total knee arthroplasty |
| Evaluation of the relationship between Orthopedic Foundation for Animals' hip joint scores and PennHIP distraction index values in dogs |
| Experimental investigation of the risk of lateral cortex fracture during valgus tibial osteotomy. |
| Factors Associated with Outcome After Subtalar Arthrodesis |
| Five- to 18-Year Follow-Up for Treatment of Trapeziometacarpal Osteoarthritis: A Prospective Comparison of Excision, Tendon Interposition, and Ligament Reconstruction and Tendon Interposition |
| Genicular Artery Embolization for Primary Knee Osteoarthritis |
| Hip Arthroscopic Surgery for Femoroacetabular Impingement: A Prospective Analysis of the Relationship Between Surgeon Experience and Patient Outcomes |
| Hip arthroscopy with initial access to the peripheral compartment provides significant improvement in FAI patients |
| Hip Arthroscopy: An Emerging Technique and Indications |
| How do I cope with pain? Let me count the ways: awareness of pain coping behaviors and relationships with depression and anxiety |
| Imaging and Treatment of Posttraumatic Ankle and Hindfoot Osteoarthritis |
| Imaging of OA - From disease modification to clinical utility |
| Implantable Shock Absorber Provides Superior Pain Relief and Functional Improvement Compared With High Tibial Osteotomy in Patients with Mild-to-Moderate Medial Knee Osteoarthritis: A 2-Year Report. |
| Improving Visualization of the Central Compartment of the Hip with Direct MR Arthrography under Axial Leg Traction: A Feasibility Study |
| Influence of lumbar intervertebral disc degeneration on the outcome of total lumbar disc replacement: a prospective clinical, histological, X-ray and MRI investigation. |
| Interobserver and Intraobserver Reliability in the Salter Classification of Avascular Necrosis of the Femoral Head in Developmental Dysplasia of the Hip. |
| Long-term Outcomes of Partial Trapeziectomy With Capsular Interposition Arthroplasty for Osteoarthritis of the Thumb Basal Joint. |
| Low-intensity pulsed ultrasound accelerates maturation of callus in patients treated with opening-wedge high tibial osteotomy by hemicallotasis |
| Malrotated Tibial Component Increases Medial Collateral Ligament Tension in Total Knee Arthroplasty |
| Management of Elderly Traumatic Ankle Arthritis with Ilizarov External Fixation. |
| Minimally invasive internal fixation of calcaneal fractures or subtalar joint arthrodesis using the Calcanail® |
| Minimum 10-year clinical and radiological follow-up of trapeziectomy with interposition or suspensionplasty for basal thumb arthritis |
| Morphologic differences in intervertebral foramina: a radiographic study of cervical spine positions in asymptomatic men |
| Morphologic features of the acetabulum. |
| Morphological divergence in the curvature of human femoral diaphyses: Tracing the central mass distributions of cross-sections. |
| MR measurement of articular cartilage thickness distribution in the hip |
| Music-induced analgesia in chronic pain: Efficacy and assessment through a primary-task paradigm |
| Patterns of the physical, cognitive, and mental health status of older individuals in a real-life primary care setting and differences in coping styles. |
| Perceptions of group music therapy among elderly nursing home residents in Taiwan |
| Performance of knee image digital analysis of radiographs of patients with end-stage knee osteoarthritis |
| Physical examination of the knee |
| Prognostic Factors in Treating Antebrachial Growth Deformities with a Lengthening Procedure Using a Circular External Skeletal Fixation System in Dogs |
| Promising Novel Biomarkers and Candidate Drugs or Herbs in Osteoarthritis: Evidence from Bioinformatics Analysis of High-throughput Data. |
| Radiographic Diagnosis of Hip Laxity in Rottweilers: Interobserver Agreement at Eight- and Twelve-Months of Age |
| Radiographic Hip Joint Phenotype of the Pembroke Welsh Corgi |
| Radiological and Clinical Results of Rotational Acetabular Osteotomy Combined with Femoral Intertrochanteric Osteotomy for Avascular Necrosis Following Treatment for Developmental Dysplasia of the Hip |
| Radiological evaluation of the relationship between caudolateral curvilinear osteophyte and joint laxity and degenerative joint disease associated with Canine Hip Dysplasia. |
| Realignment-lengthening osteotomy for malunited distal fibular fracture |
| Repair of a large osteochondral defect in the knee joint using autologous and artificial bone graft combined with motion preserving distraction arthroplasty: a case report |
| Repair of large full-thickness articular cartilage defects in the rabbit - The effects of joint distraction and autologous bone-marrow-derived mesenchymal cell transplantation |
| Role of physical activity in cartilage damage progression of subjects with baseline full-thickness cartilage defects in medial tibiofemoral compartment: data from the Osteoarthritis Initiative |
| Salvage Options for Flexor Carpi Radialis Tendon Disruption During Ligament Reconstruction and Tendon Interposition or Suspension Arthroplasty of the Trapeziometacarpal Joint |
| Soft-Tissue Balancing in Total Knee Arthroplasty: Cruciate-Retaining Versus Posterior-Stabilized, and Measured-Resection Versus Gap Technique |
| Supramalleolar Osteotomy vs Arthrodesis for the Treatment of Takakura 3B Ankle Osteoarthritis |
| Surgical management for primary osteoarthritis of the elbow |
| The Best Position of Bone Grafts in the Medial Open-Wedge High Tibial Osteotomy: A Finite Element Analysis |
| The Disproportionate Increase of the Intraoperative Flexion and Extension Gap Space after Posterior Cruciate Ligament Resection in Total Knee Arthroplasty. |
| The effect of high, medium and low mobilization forces applied during a hip long-axis distraction mobilization on the strain on the inferior ilio-femoral ligament and psoas muscle: A cadaveric study |
| The effect of joint distraction osteogenesis combined with platelet-rich plasma injections on traumatic ankle arthritis |
| The Effect of Scapular Fixation on Scapular and Humeral Head Movements during Glenohumeral Axial Distraction Mobilization |
| The medial gap is a reliable indicator for intraoperative soft tissue balancing in posterior-stabilized total knee arthroplasty |
| The postoperative patella height: a comparison of additive and subtractive high tibial osteotomy in correcting the genu varum |
| The Use of Platelet-Rich Plasma in Symptomatic Knee Osteoarthritis |
| The value of Weight-Bearing CT scan in the evaluation of subtalar distraction bone block arthrodesis: Case report |
| Thumb Carpometacarpal Arthritis |
| Transtrochanteric rotational osteotomy for various hip disorders |
| Treatment of advanced carpometacarpal joint disease: Carpometacarpal arthroplasty with ligament interposition |
| Triple Pelvic Osteotomy and Double Pelvic Osteotomy |
| Ultrasound measurement of the effects of high, medium, and low hip long-axis distraction mobilization forces on the joint space width and its correlation with the joint strain |
| Ultrasound-guided platelet-rich plasma injections for the treatment of common peroneal nerve palsy associated with multiple ligament injuries of the knee. |
| Using real-time ultrasound imaging as adjunct teaching tools to enhance physical therapist students' ability and confidence to perform traction of the knee joint |
| Which physical examination tests provide clinicians with the most value when examining the shoulder? Update of a systematic review with meta-analysis of individual tests |
| Yoga for Osteoarthritis Nursing and Research Considerations |
